# When environments mismatch—the limits of transgenerational climate memory in Scots pine

**DOI:** 10.1093/treephys/tpag141

**Published:** 2026-09-23

**Authors:** Na Luo, Arthur Gessler, Falko Noé, Igor A Yakovlev, Isabel Weirich, Bernd Kammerer, Rie Shimizu-Inatsugi, Stefan Hunziker, Simon Lagies, Simone M Pieber, Daniel Mohl, Ugo Molteni, Kentaro Shimizu, Arun K Bose

**Affiliations:** WSL Swiss Federal Institute for Forest, Snow and Landscape Research, Zürcherstrasse 111, CH-8903 Birmensdorf, Switzerland; WSL Swiss Federal Institute for Forest, Snow and Landscape Research, Zürcherstrasse 111, CH-8903 Birmensdorf, Switzerland; Department of Environmental Systems Science, ETH Zurich, Institute of Terrestrial Ecosystems (ITES), Universitätstrasse, 22, 8092 Zurich, Switzerland; Department of Health Sciences and Technology, ETH, Laboratory of Regenerative and Movement Biology, Schwerzenbach, Zurich 8603, Switzerland; Functional Genomics Center Zurich, ETH Zurich and University of Zurich, Zurich 8057, Switzerland; Department of Molecular Plant Biology, Norwegian Institute of Bioeconomy Research, 1431 Ås, Norway; WSL Swiss Federal Institute for Forest, Snow and Landscape Research, Zürcherstrasse 111, CH-8903 Birmensdorf, Switzerland; Faculty of Biology, University of Freiburg, Schänzlestraße 1, 79104 Freiburg, Germany; Core Competence Metabolomics, University of Freiburg, Habsburgerstraße 49, 79104 Freiburg, Germany; Department of Evolutionary Biology and Environmental Studies, University of Zürich, Winterthurerstrasse 190, 8057 Zurich, Switzerland; WSL Swiss Federal Institute for Forest, Snow and Landscape Research, Zürcherstrasse 111, CH-8903 Birmensdorf, Switzerland; Core Competence Metabolomics, University of Freiburg, Habsburgerstraße 49, 79104 Freiburg, Germany; WSL Swiss Federal Institute for Forest, Snow and Landscape Research, Zürcherstrasse 111, CH-8903 Birmensdorf, Switzerland; Faculty of Biology, University of Freiburg, Schänzlestraße 1, 79104 Freiburg, Germany; WSL Swiss Federal Institute for Forest, Snow and Landscape Research, Zürcherstrasse 111, CH-8903 Birmensdorf, Switzerland; Department of Evolutionary Biology and Environmental Studies, University of Zürich, Winterthurerstrasse 190, 8057 Zurich, Switzerland; Kihara Institute of Biological Research, Yokohama City University, Japan; WSL Swiss Federal Institute for Forest, Snow and Landscape Research, Zürcherstrasse 111, CH-8903 Birmensdorf, Switzerland; Forestry and Wood Technology Discipline, Khulna University, Khulna, Bangladesh

**Keywords:** epigenetic, metabolomics, plant growth, plant physiology, transcriptome, transgenerational acclimation

## Abstract

A long-term irrigation experiment in the dry Rhône Valley of Switzerland provides a unique opportunity to examine transgenerational environmental memory effects of persistent drought and irrigation in Scots pine (*Pinus sylvestris* L.). Previous work in this system showed that offspring from mother trees exposed to chronic drought (non-irrigated control) versus long-term irrigation differed markedly in growth and survival. Offspring from irrigated mothers grew faster under irrigated conditions but performed poorly under drought, whereas offspring from drought-exposed mothers showed higher growth and survival under drought conditions. While these responses suggested transgenerational environmental memory, the relative contributions of maternal provisioning versus epigenetic mechanisms could not be fully resolved. Notably, mother trees did not differ in genotype, and seed mass did not vary between treatments, indicating a limited role of maternal effects. Here, we tested whether transgenerational environmental memory effects persist after offspring were grown for 3 years under non-experimental conditions before being re-exposed to experimental hot-drought and irrigation treatments. In contrast to the earlier study, offspring from irrigated mothers did not show higher growth performance under irrigated conditions, indicating that irrigation-related memory weakened during the intervening non-treated phase. Conversely, seedlings originating from drought-exposed mothers consistently exhibited higher carbon assimilation and stem growth than those from irrigated mothers, irrespective of experimental treatment. Gene expression analyses showed differences in the number and identity of differentially expressed genes between offspring of drought- and irrigation-exposed mothers, although we lack data on DNA methylation, histone modifications or small RNAs. Patterns in primary metabolites broadly mirrored the results of gene expression, with modest differences in osmoprotectants and stress-related compounds. Overall, our results indicate that transgenerational environmental memory effects in *P. sylvestris* can diminish over time and, depending on the parental and offspring environments, may even result in mismatch and maladjustments.

## Graphical Abstract

**Graphical Abstract:**
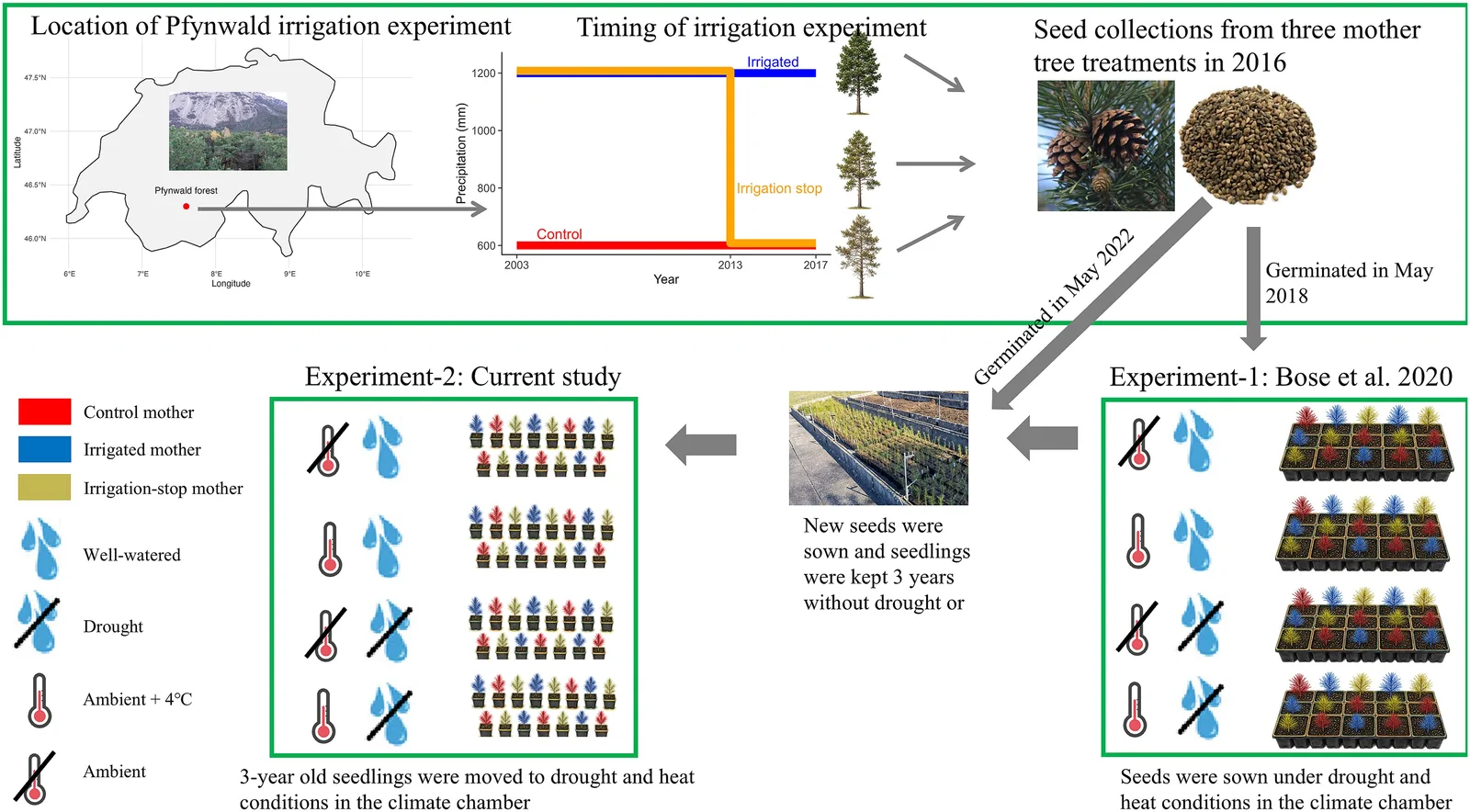

## Introduction

Extreme climatic events such as droughts and heat waves are increasing worldwide (Spinoni et al. 2014), particularly in Central and Mediterranean Europe, which has experienced several megadroughts over the past two decades (Schuldt et al. 2020, Chen et al. 2025). Droughts limit the water supply for trees, trigger stomatal closure, downregulate photosynthesis and cause canopy dieback, hydraulic failure and tree mortality (Sala et al. 2010, Anderegg et al. 2012, Ruehr et al. 2019, Bose et al. 2024). Due to the long generation times of most tree species, evolutionary adaptation occurs slowly (Kremer et al. 2012). However, under rapid environmental change, trees may respond through phenotypic plasticity, i.e., environmentally induced changes in phenotype within an individual’s lifetime (Limousin et al. 2012, Valladares et al. 2014, Bachofen et al. 2021, Lemaire et al. 2021). Several studies have reported phenotypic adjustments of trees under persistent abiotic and biotic stresses (e.g., Pritzkow et al. 2021, Balestrini et al. 2022, Encinas-Valero et al. 2022). Such environmentally induced phenotypic adjustments can even be transmitted to the following generation, whereby parental environmental conditions influence offspring phenotype, growth and survival (Yakovlev et al. 2010, Raj et al. 2011, Gömöry et al. 2017, Bäurle and Trindade 2020, Perrella et al. 2022).

On a mechanistic level, maternal and epigenetic effects can lead to such transgenerational environmental memory effects (Holeski et al. 2012, Alonso et al. 2019, Miryeganeh and Armitage 2025). Maternal effects refer to the direct influence of the maternal genotype on progeny fitness, conveyed through factors such as seed size or nutrient provision to the embryo (Holeski et al. 2012). For example, seed mass has been identified as a major determinant of seedling growth in Scots pine (Castro 1999). Epigenetic effects are defined as heritable changes in phenotype that occur through mechanisms including DNA methylation, histone modification and small RNAs, which can alter the regulatory states of genes or genomic regions (Bossdorf et al. 2008, Sow et al. 2021, Miryeganeh and Armitage 2025). A recent study revealed that plant responses to high CO_2_ levels are heritable and mediated by DNA methylation (Panda et al. 2023). Environmental factors such as drought and high temperature can alter DNA methylation patterns and induce other epigenetic modifications (Raj et al. 2011, Klupczyńska and Ratajczak 2021). Drought-induced changes in DNA methylation have been documented in several tree species, including *Quercus ilex* (Rico et al. 2014), *Populus* species (Raj et al. 2011, Sow et al. 2021) and in *Fagus sylvatica* (Hrivnák et al. 2017). Such epigenetic modifications can regulate gene expression and thereby influence cellular functions and phenotypic traits, including tolerance to heat and drought stress, as shown in *Pinus radiata* (Lamelas et al. 2024).

At the molecular level, it is known that epigenetic mechanisms, including DNA methylation, histone modifications and small RNA interference, are inherently reversible (Bräutigam et al. 2013). Plants tend to prioritize growth and reproduction under favorable conditions by resetting or ‘forgetting’ stress-induced epigenetic marks, a phenomenon referred to as epigenetic forgetfulness (Crisp et al. 2016, Staacke et al. 2025). This strategy may enhance plant fitness when environmental conditions improve by allowing plants to reset or reduce stress-induced transgenerational effects. Consequently, the influence of transgenerational plasticity may weaken or disappear over time, particularly when environmental conditions differ across generations (Hilker and Schmülling 2019). Nevertheless, evidence for the persistence of long-term transgenerational plasticity, particularly beyond early developmental stages, is emerging. Several studies have shown that transgenerational effects can persist beyond germination and seedling establishment and influence later stages of the plant life cycle, including responses to both abiotic and biotic stresses (e.g., Vivas et al. 2013, Walter et al. 2016, Sandner et al. 2018, Sobral et al. 2021, Ramos-Muñoz et al. 2024).

Whether such long-lasting transgenerational effects persist under chronic environmental stress in natural populations, however, remains poorly understood. In the Rhône valley (Switzerland), a persistently drought-exposed Scots pine forest has been subjected to a long-term irrigation experiment since 2003. Unlike many drought experiments that impose water limitation through rainfall exclusion or warming (e.g., Adams et al. 2015, Gavinet et al. 2019, Grams et al. 2021), our study site lies at the extremely dry distribution edge of Scots pine in the inner Alpine Rhône valley, where trees experience recurrent chronic drought stress and mortality (Rigling et al. 2013), indicating that current climatic conditions already impose strong water limitation. The irrigation treatment alleviates chronic drought stress by approximately doubling growing-season precipitation. This design allows us to assess both the long-term memory of natural drought (hereafter, control) the recovery trajectories following ‘irrigation’ treatment (Dobbertin et al. 2010).

In the earlier study, Bose et al. (2020) reported that under controlled conditions, offspring from trees regularly exposed to drought exhibited greater tolerance to hot-drought conditions than offspring from long-term irrigated trees in the year of their germination. However, under high water supply and full sunlight, offspring originated from trees exposed to drought showed reduced growth and were outperformed by the progeny of irrigated trees. These findings highlight the critical role of transgenerational environmental memory effects in shaping tree responses to environmental conditions and support the concept of a trade-off between stress resistance and growth (Volaire et al. 2023). However, important questions remain unanswered: How long will this transgenerational environmental memory persist and continue to influence offspring responses? Is it mainly helping to adjust seedlings’ traits and functioning in the very first phase of development, or might it partially compensate on a longer timescale for the slow adaptation potential of trees in a rapidly changing climate? Knowledge of these points will aid better understanding of the future resilience (or the lack thereof) of forest ecosystems and to adjust management practices.

In the current study, we investigated whether environmental memory inherited from irrigation- and drought-exposed mother trees remained significant in shaping the functional responses of Scots pine seedlings after 3 years of growth under common, non-treated environmental conditions. Unlike studies focusing exclusively on transgenerational effects under matching parental and offspring environments (Marshall and Uller 2007, Burgess and Marshall 2014), our experimental design included both matching stress conditions (drought) and an additional offspring treatment (warming), reflecting climatic conditions that have become increasingly frequent in recent decades. This approach allowed us to assess not only the persistence of transgenerational environmental memory under matching conditions, but also how current environmental conditions interact with parental environmental effects.

We examined a range of functional responses, including biomass growth, physiological performance, primary metabolome profiles and gene expression patterns. Previous work from other plant systems suggests that transgenerational environmental memory effects on phenotype may weaken over time when offspring are no longer exposed to the same environmental conditions experienced by the parental generation. Therefore, we hypothesized that the transgenerational environmental memory effects observed during germination and early seedling development would diminish after 3 years. More specifically, we expected a phenotypic convergence among progenies from drought- and irrigation-exposed mother trees, such that differences in growth and physiological traits would become smaller over time under shared environmental conditions. At the same time, we anticipated that signatures of transgenerational environmental memory would remain detectable at the molecular level, particularly in the metabolome and transcriptome, even if phenotypic differences weakened. This hypothesis of partial dissociation between phenotypic and molecular memory is consistent with recent studies showing that stress-induced epigenetic and transcriptomic signatures can persist across generations after visible phenotypic acclimation has faded (Crisp et al. 2016, López et al. 2024). Our study provides valuable insights into the dynamics of transgenerational memory in trees, particularly how such memory evolves over time and influences offspring responses when environmental conditions shift. By integrating physiological, metabolic and molecular analyses, we aimed to deepen our understanding of the persistence and mechanisms of ecological memory in Scots pine and its implications for long-term tree adaptation to changing environments.

## Materials and methods

### Study site

The study was conducted at a long-term irrigation experiment established in 2003 in the Pfynwald forest (46°18′N, 7°36′E; 615 m a.s.l.), located in the dry inner-Alpine Rhone Valley, Switzerland. The site is characterized by warm and dry conditions, with a mean annual temperature of 10.6°C and annual precipitation of 576 mm (based on observations from the nearest climate station), shallow rocky soils with low water-holding capacity, and recurrent drought events, placing Scots pine (*Pinus sylvestris* L.) near its dry distribution limit (Bose et al. 2022).

### Irrigation experiment

The experiment was established in an even-aged Scots pine stand (~100 years old) and consists of eight plots (1000 m^2^ each) arranged in a randomized block design*.* Since 2003, four plots have been irrigated during rainless nights throughout the growing season (April–September) using a sprinkler system, approximately doubling natural precipitation, while four plots have remained non-irrigated. In 2013, the irrigated plots were further divided into two treatments: continued irrigation and ‘irrigation-stop’ (no irrigation since October 2013). The long-term irrigation treatment alleviates chronic soil moisture limitation and drought stress at the site, whereas the non-irrigated plots represent the ambient climatic conditions currently experienced by forests in this region of Switzerland. These ambient conditions are characterized by increasing atmospheric evaporative demand and recurrent drought events, making the non-irrigated plots representative of the prevailing drought-prone conditions in the area (Bose et al. 2022, Hunziker et al. 2022).

### Seed genotyping

Seeds from 57 randomly selected mother trees (23 irrigated, 18 irrigation-stop and 16 control mother trees) were sampled in February 2017 when cones were ripe (Bose et al. 2020). The results from seed genotyping showed no significant genetic differentiation among the three maternal treatments (irrigated, irrigation-stop and control). Analysis of molecular variance indicated that only 1% of the total genetic variation occurred among treatments, with 99% found within treatments. Consistently, genetic structure analysis revealed a single genetic cluster across all seeds. In addition, the seeds from the different treatments did not differ in weight (Bose et al. 2020).

### Analyses of seeds

Seeds collected in February 2017 from the same mother trees were used in the present experiment. Seeds were extracted from opened cones, and filled seeds were separated from empty seeds using a blower before being stored at 2 °C (He et al. 2018). Carbon (C) and nitrogen (N) contents and the isotope composition of C (δ^13^C) and N (δ^15^N) were quantified from 1 ± 0.1 mg of finely ground and homogenized dried seed material weighed into tin capsules using an elemental analyzer (Euro EA; Hekatech GmbH, Wegberg, Germany) coupled to an Isotope Ratio Mass Spectrometer (IRMS Delta V Advantage; Thermo Scientific, Bremen, Germany). Seeds were randomly selected from the three origins of mother trees (irrigated, irrigation-stop and control). All samples’ isotopic ratios were denoted using the δ notation (%) relative to Vienna Pee Dee Belemnite (VPDB) for C and N_2_ in air for N. The measurement accuracy was ~0.1% for δ^13^C and δ^15^N.

### Seedlings

From each of the three mother tree groups, randomly selected seeds were sown under greenhouse conditions in Birmensdorf in May 2019. Germinated seedlings were kept outside of the greenhouse under natural conditions for a 3-year period, until the end of May 2022. The seedlings were monitored daily throughout the growing season, and irrigation was applied whenever the soil became sufficiently dry to prevent drought stress. Thus, irrigation was not applied according to a fixed schedule or at a predefined frequency, but rather as needed based on soil moisture conditions (see Figure 1). The seedlings were then transferred to climate chambers on 1 June 2022. Two temperature (ambient temperature representative of Pfynwald and ambient +4 °C) and two watering treatments (intermittent drought and well-watered) were applied on 1 July 2022*,* comparable to the treatments detailed by Bose et al. (2020). Soil volumetric water content (VWC) was measured two to three times per week to monitor the temporal development of the watering treatments. The intermittent drought treatment targeted a VWC of ~10%, whereas VWC in the well-watered treatment was generally maintained above 20% (see Figure S1 available as Supplementary Data at *Tree Physiology* Online).

**Figure 1:**
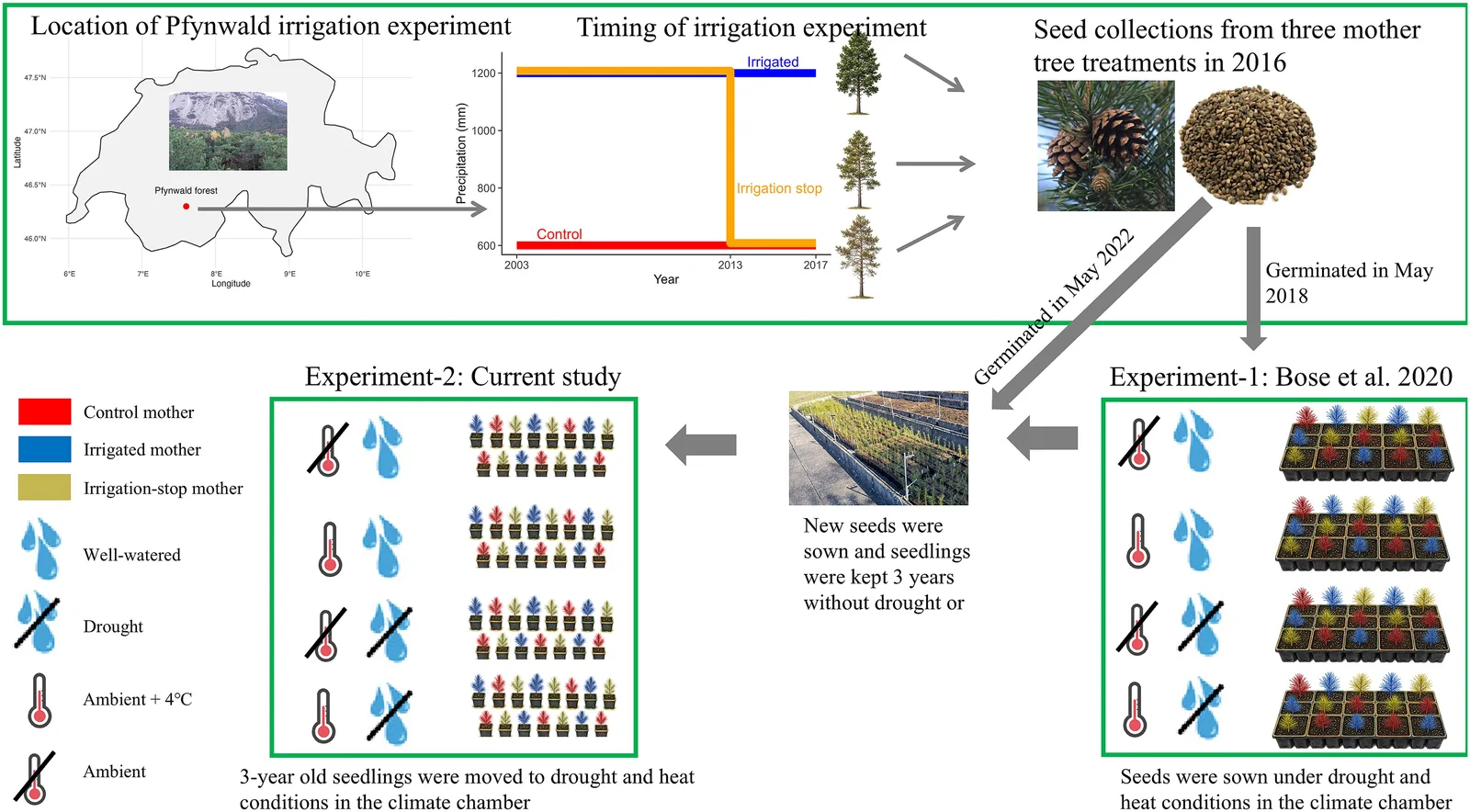
Conceptual diagram illustrating the experimental design and historical development of the study. The first experiment (Bose et al. 2020) examined growth and survival of Scots pine progeny as a function of maternal tree origin, drought and heating treatments. The second experiment (current study) investigated whether the memory effects identified by Bose et al. (2020) persist when progeny is maintained under non-treated conditions for 3 years prior to re-exposure to the treatment conditions. In the second experiment, 3-year old seedlings were transferred to climate chambers. Two temperature (ambient temperature and ambient +4 °C) and two watering treatments (intermittent drought and well-watered) were applied, comparable to the treatments of Bose et al. (2020).

There were three chamber replicates per temperature treatment. Consequently, there were four different treatments for each of the three mother tree groups, resulting in 12 different groups in total. Light intensity was controlled and maintained at comparable levels across all chambers to avoid chamber-specific light effects.

The experiment followed the natural seasonal photoperiod at the experimental location. Accordingly, day length decreased from ~15 h 53 min at the beginning of the experiment to 11 h 21 min at the end of the experiment, while the corresponding dark period increased from ~8 h 07 min to 12 h 39 min. Air temperature was recorded at 10-min intervals in each of the six climate chambers throughout the experiment. Daylight and dark periods were defined separately for each date using the corresponding local sunrise and sunset times. Mean air temperatures during the natural daylight and dark periods were 22.3 °C and 17.7 °C, respectively, in the three ambient chambers, and 25.7 °C and 20.6 °C, respectively, in the three heated chambers. Daily mean air temperatures for the individual chambers are presented in Figure S2 available as Supplementary Data at *Tree Physiology* Online.

### Data collection and analysis

#### Seedling physiology

We used two replicated individuals (seedlings) for each of the treatment combinations. Stomatal conductance (g_s_) refers to the conductance of stomata to water vapor diffusion from the leaf interior to the atmosphere. In this study, g_s_ was obtained directly from the gas-exchange system LI-COR 6800 and is expressed as mol H₂O m^−2^ s^−1^, which was measured in mid-August 2022 (1.5 months after the start of the treatment) and mid-September 2022 (2.5 months after the start of the treatment). Light-saturated assimilation (A_sat_) and intrinsic water-use efficiency (WUE_i_, A_sat_/g_s_) were obtained and calculated using point measurements at a mixing ratio of 400 p.p.m. CO_2_.

The measurements were conducted at a photosynthetic photon flux density of 1500 μmol m^−2^ s^−1^ (light saturation), an air temperature of 25 °C inside the cuvette, and a vapor pressure deficit (VPD) of 1.5 kPa. The measurements were performed on mature and healthy current-year needles, chosen from the upper canopy of each seedling, between 9:00 a.m. and 5:00 p.m. All seedlings were measured under control and relatively stable conditions (climate chambers), ensuring that temperature and light conditions remained constant throughout the measurement period. In addition, seedlings were randomly assigned to measurement order to avoid systematic bias associated with time of day.

Approximately six needles were enclosed in a 2 cm^2^ cuvette stabilized with tape. The enclosed needles were harvested after each measurement. The needle area was measured using a flatbed scanner (Epson Perfection V800 scanner) with an image software (silverfast 8.8), and the area was analyzed using Pixstat v1.3 (Schleppi 2018). The recorded gas exchange values were corrected using the projected needle area. Whole-seedling photosynthesis was estimated by multiplying area-based A_sat_ by the total needle area of each individual, which was determined destructively by harvesting all needles and scanning them to calculate needle area.

For determining non-structural carbohydrates (NSC) consisting of soluble sugars and starch, entire plants from all treatments were harvested at the beginning of treatments (3 July 2022), and at the end of the growing season when the treatments ended (conducted on 10 October 2022). We harvested six to seven replicate individuals for each treatment × seed-type combination during each sampling campaign and subsequently measured their NSC compounds*.* Washed coarse and fine roots, bark, stem (debarked wood), new needles (current year) and old needles (older than 1 year) were separated and dried at 60 °C for at least 72 h. They were weighed and finely ground into a powder using a ball mill (MM400 ball mill; Retsch GmbH, Haan, Germany). The NSC were determined separately for each organ using a modified procedure based on Wong’s methodology (Wong 1990) and adjustments outlined by Hoch et al. (2002). We define NSC as low-molecular-weight sugars (glucose, fructose and sucrose) and starch. We took 10–12 mg of finely ground material from all organs from both the harvests and placed them into 2 mL of distilled water, then subjected them to steam boiling for 30 min. After centrifugation, we treated a 200 μL portion with invertase (Product Code: I4504-250MG, Sigma-Aldrich) and isomerase (Product Code: P5381-5KU, Sigma-Aldrich) derived from baker’s yeast (*Saccharomyces cerevisiae*), to hydrolyze sucrose into glucose and convert fructose concurrently to glucose. Total glucose content (i.e., sugars) was measured at a wavelength of 340 nm using a 96-well microplate photometer (HR 7000 Microplate Photometer; Hamilton Company, Reno, NE, USA). This measurement followed enzymatic conversion to gluconate-6-phosphate through the hexokinase reaction, enabled by adding hexokinase (Product Code: G3293-20ML, Sigma-Aldrich). To determine the total non-structural carbohydrate (TNC) content, we incubated 500 μL of the extract (which includes sugars and starch) at 49 °C with fungal amyloglucosidase sourced from Aspergillus niger (Product Code: 10115-5G-F, Sigma-Aldrich) for 15 h. This process caused the transformation of starch into glucose. The photometric method described earlier was used to quantify the total glucose content (equivalent to TNC). Starch content was determined by subtracting the number of free sugars from TNC. Calibration was performed using pure solutions of starch, glucose, fructose and sucrose as standard references. To ensure extraction consistency, standard plant powder (Orchard leaves; Leco, St Joseph, MI, USA) was also included.

The NSC concentrations are based on a percentage of dry matter. Uniform laboratory setting and unaltered protocols were consistently employed throughout the sample processing phase, thereby effectively circumventing any potential problems related to inter-method or inter-laboratory comparisons, as reported by Quentin et al. (2015). Relative sugar content is given as a ratio to total nonstructural carbohydrates.

#### Stem growth

The stem volume was calculated using the following equation:


$$ stem\ volume\ (SV)\kern0.5em =\frac{1}{3}\bullet \pi \bullet height\bullet{\left(\ \frac{diameter}{2}\right)}^2 $$

Stem volume growth was calculated as the difference between stem volume measured on 7 October 2022, at the end of the treatment periods, and on 3 July 2022 at the start of the treatments. Stem diameter at the root collar (ground level) and height of the seedlings were measured using a digital caliper and tape, respectively. Primary shoot elongation in Scots pine occurs mainly during spring (May–June); therefore, because our treatment period extended from July to September, we focused on secondary (radial) stem growth, which more accurately captured treatment effects during the experimental period.

#### Primary metabolites

Current-year needles were collected on 30 August 2022, i.e., after ~2 months of drought and heat treatments. From each of the six climate chambers, two seedlings of each drought treatment were randomly chosen for needle sampling. Six replicates were sampled for each of the 12 groups (i.e., 6 replicated seedlings × 3 levels of mother tree × 2 levels of watering × 2 levels of temperature = 72 samples). After the harvest, the needles were immediately frozen in liquid nitrogen. The samples were then kept in the freezer at −80 °C until analysis. For the chemical analysis, several needles were pulverized in a mortar for each sample by pouring liquid nitrogen over them and then grinding with a pestle. Metabolite extraction, derivatization and non-target analysis was then performed with GC–MS (Gas Chromatography–Mass Spectrometry) according to Jansen et al. (2014) and Hunziker et al. (2024). While some compounds overlap with the NSC analysis, the two datasets are not directly interchangeable. The NSC data reflects carbon storage capacity across organs, while the primary metabolite data reflects the metabolic state and stress responses of needle tissue. The primary metabolic profile is meant to provide a functional linkage between gene expression and phenotypical traits.

#### Gene expression analysis

Current-year needles were collected on 30 August 2022, for transcriptomic analyses with the following sampling design: 3 replicated seedlings × 3 levels of mother tree × 2 levels of watering × 2 levels of temperature = 36 samples. The needle tissues were frozen in liquid nitrogen immediately after the collection and stored at −80 °C. RNA was extracted with the RNeasy Plant Mini Kit (Qiagen), and the concentration was measured by Qubit using the RNA BR Assay Kit (Thermofischer Scientific). The mRNA library was prepared from the 500 ng of the total RNA samples using the Illumina Truseq stranded mRNA library kit (Illumina, Cat. No. RS-122-2301) and sequenced by the Novaseq 6000 System (Illumina). The RNA extraction of two samples out of 36 was not successful and they were excluded from the sequencing and further analyses. One sample originated from a control mother tree and the other from an irrigated mother tree; both were subjected to the ambient temperature and drought treatments.

The raw reads were first cleaned by removing adapter sequences and poly-x sequences (>9 nt used for detection) using fastp (Version 0.20.0) (Dobin et al. 2013). Sequence alignment of the resulting high-quality reads to the *Pinus taeda* reference genome (build Pita v2.01) was performed using STAR 2.7.10b and quantification of gene-level expression (gene model definition from Pita v2.01 TreeGenes) was carried out using featureCounts implemented within Rsubread (Version 2.12.2) (Liao et al. 2014).

### Statistical analysis

Linear mixed-effect models (Zuur et al. 2009) were used to analyze growth, physiological and metabolite-related variables. Each response variable was modeled as a function of mother tree treatment (three levels: control, irrigation, irrigation-stop), drought treatment (two levels: drought and watered) and temperature treatment (two levels: ambient and ambient +4 °C). Preliminary analyses indicated that neither two-way nor three-way interaction terms were significant; these were therefore excluded from the final models. We used multiple comparison ‘Tukey’ tests for pairwise differences across mother tree treatments. These analyses were executed using the function ‘*emmeans*’ of the R package ‘*emmeans*’ (Lenth 2024).

The hierarchical structure of the experiment (i.e., seedlings nested within climate chambers) was accounted for by including random effects. Growth and physiological variables were square root transformed to meet model assumptions. Models were fitted using the *lme* function in the *nlme* package in R (Pinheiro et al. 2014, R Development Core Team 2022). Assumptions of normality and homogeneity of variance were assessed visually using residual diagnostics.

We performed transcriptomic analysis to estimate the influence of mother tree treatments on the transcriptomes under different stress conditions. We expected to detect differences in the transcriptomes of seedlings from irrigated and irrigated-stop mother trees in response to drought and well-watered conditions under ambient and high temperatures. For detecting the differentially expressed genes, we used the *glm* approach implemented in the software package DESeq2 (R version: 4.2.2, DESeq2 version: 1.38.0) (Love et al. 2014). Genes showing altered expression with *P*-value ≤0.05 and log_2_ ratio >1 were considered significant. Over-representation analysis (ORA) was conducted based on differentially expressed genes with *P*-value <0.05 using clusterProfiler (Version 4.6.0) (Wu et al. 2021). Terms with a false-discovery rate <0.05 were considered significant.

## Results

### Seeds

In seeds, the concentrations of C and N, and δ^15^N did not vary significantly among the different origins of the mother trees (control, irrigation and irrigation-stop). However, the δ^13^C levels were significantly lower in the seeds from irrigated mother trees compared with those from control mother trees (Table 1).

**Table 1:** Effect of irrigation treatments (control, irrigated and irrigation-stop) on δ^13^C, δ^15^N, N and C concentration in seeds. All values are means ±SE, and *F*-values associated with *P-*values (in the bracket) are provided. Bold *P*-values indicate significant treatment differences. Seeds from 57 randomly selected mother trees (23 irrigated, 18 irrigation-stop and 16 control mother trees) were sampled in February 2017.

Seed type	δ^13^C	δ^15^N	N (%)	C (%)
Control	−24.03 ± 0.12	−5.41 ± 0.18	5.7 ± 0.15	53.29 ± 0.35
Irrigated	−25.02 ± 0.18	−5.75 ± 0.16	5.91 ± 0.09	53.22 ± 0.18
Irrigation-stop	−23.83 ± 0.11	−5.42 ± 0.15	5.62 ± 0.08	53.44 ± 0.52
Statistical tests, *F*-values (*P-*values)	47.24 (**<0.001**)	1.63 (0.442)	4.49 (0.106)	0.18 (0.914)

### Seedling growth and physiology

Seedling-level assimilation rate was lower in the offspring of irrigated mother trees compared with those from control mother trees, regardless of drought and heat treatments (Table 2 and Figure 2). Drought and heat treatments significantly decreased stomatal conductance while increased WUEi, irrespective of mother tree origins (Table 2 and Figure 2). The drought treatment significantly reduced the assimilation rate by 14.8%, whereas warming produced only a nonsignificant 0.9% reduction (Table 2).

**Table 2:** Results (coefficients ± standard errors and associated *P*-values) from the statistical analyses for all growth and physiological variables. Linear mixed-effects models were applied, with each response variable modeled as a function of mother tree treatments (three levels: Control, irrigation, irrigation-stop), drought treatments (two levels: Drought and watered), and temperature treatments (two levels: Ambient and ambient +4 °C). Preliminary analyses indicated that neither two-way nor three-way interaction effects were significant and were therefore excluded from the final models. Pairwise comparisons and corresponding *P*-values were obtained using post-hoc Tukey multiple comparison tests. Bold *P*-values indicate statistically significant effects.

Variables	Mother tree treatment			Drought vs watered	Ambient heat vs ambient +4 °C
	Control vs irrigation	Control vs irrigation-stop	Irrigation vs irrigation-stop		
Stomatal conductance (mol m^−2^ s^−1^)	0.028 ± 0.015 (0.150)	0.022 ± 0.015 (0.288)	−0.005 ± 0.015 (0.926)	−0.03 ± 0.01 **(0.004)**	−0.04 ± 0.01 **(0.04)**
Assimilation (μmol m^−2^ s^−1^)	**0.226 ± 0.088** **(0.028)**	0.151 ± 0.087 (0.195)	−0.075 ± 0.086 (0.659)	−0.16 ± 0.07 **(0.02)**	−0.009 ± 0.09 (0.93)
WUEi (μmol mol ^−1^)	−0.118 ± 0.345 (0.937)	−0.230 ± 0.342 (0.780)	−0.112 ± 0.340 (0.942)	0.67 ± 0.28 **(0.02)**	1.31 ± 0.28 **(0.01)**
Area of current year needles (cm^2^)	**1.149 ± 0.305** **(0.001)**	**1.319 ± 0.306** **(0.000)**	0.169 ± 0.302 (0.840)	−0.67 ± 0.25 **(0.01)**	0.09 ± 0.25 (0.73)
Diameter growth (cm)	**0.092 ± 0.038** **(0.045)**	0.041 ± 0.041 (0.581)	−0.052 ± 0.039 (0.386)	0.02 ± 0.03 (0.58)	0.10 ± 0.06 (0.16)
Volume growth (cm^3^)	**0.055 ± 0.027** **(0.014)**	0.037 ± 0.028 (0.408)	−0.018 ± 0.027 (0.782)	0.003 ± 0.02 (0.88)	0.07 ± 0.04 (0.14)
NSC in new needles (%)	0.621 ± 0.509 (0.452)	1.076 ± 0.509 (0.106)	0.454 ± 0.514 (0.655)	**−0.621 ± 0.249** **(0.017)**	−0.751 ± 0.417 (0.147)
NSC in stem (%)	−0.438 ± 0.313 (0.354)	0.439 ± 0.329 (0.389)	**0.877 ± 0.316** **(0.025)**	−0.331 ± 0.235 (0.168)	−0.515 ± 0.401 (0.269)
NSC in bark (%)	−0.366 ± 0.216 (0.225)	0.364 ± 0.220 (0.241)	**0.730 ± 0.216** **(0.006)**	−0.223 ± 0.172 (0.199)	−0.320 ± 0.307 (0.357)
NSC in fine roots (%)	−0.182 ± 0.153 (0.470)	−0.152 ± 0.155 (0.594)	0.030 ± 0.156 (0.980)	−0.197 ± 0.85 **(0.025)**	0.166 ± 0.211 (0.478)
NSC in coarse roots (%)	−0.097 ± 0.218 (0.897)	0.342 ± 0.220 (0.283)	0.439 ± 0.220 (0.133)	−0.362 ± 0.159 **(0.027)**	0.007 ± 0.186 (0.971)
NSC in whole plant (%)	−0.546 ± 0.611 (0.648)	1.120 ± 0.613 (0.181)	**1.666 ± 0.612** **(0.030)**	−0.812 ± 0.399 (0.053)	−0.590 ± 0.501 (0.305)

**Figure 2:**
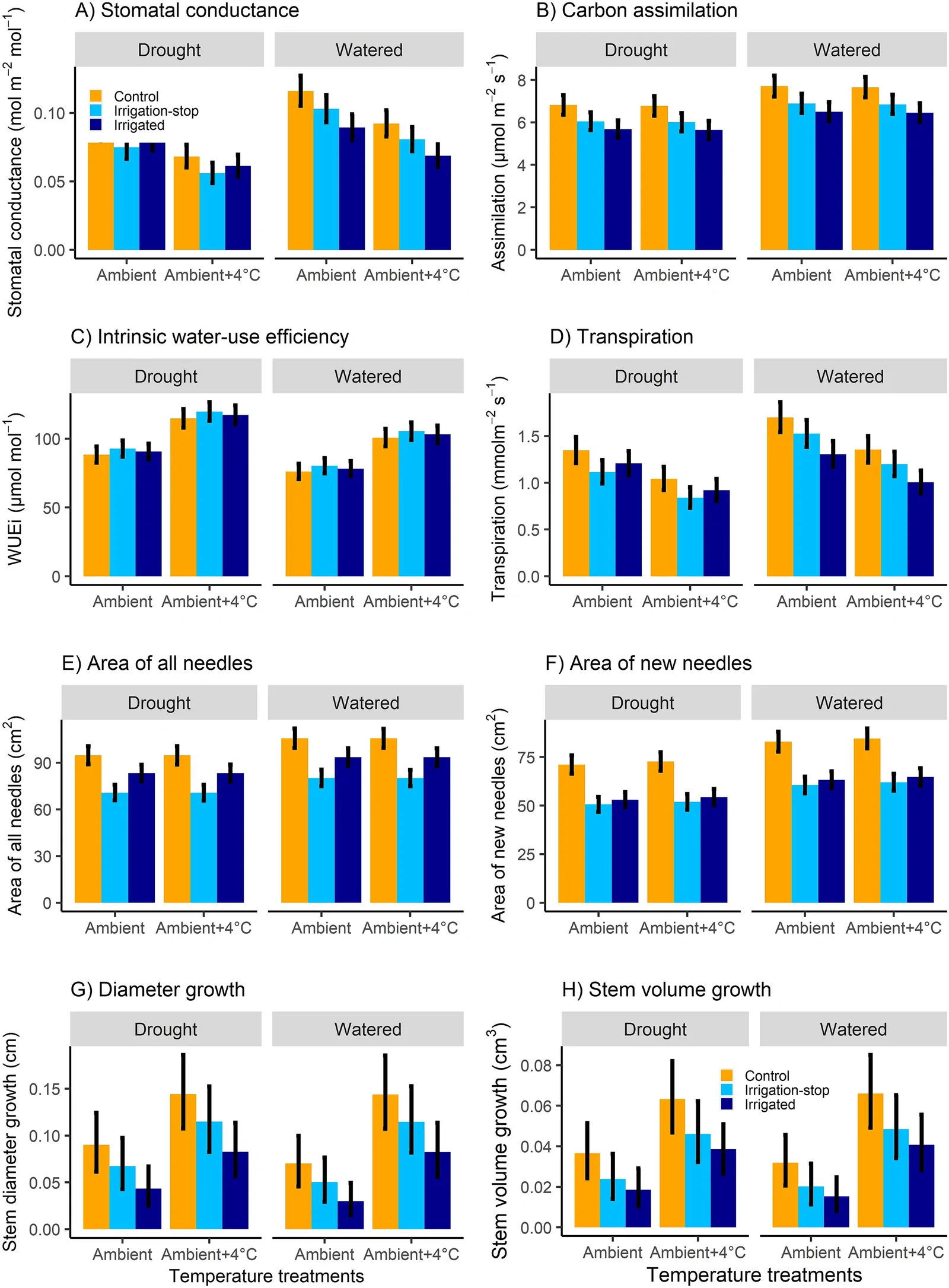
Phenotypic differences among offspring originated from three mother tree origins (control, irrigated and irrigation-stop) and exposed to two treatment conditions (heat and drought). The irrigation memory of mother trees induced a decline of stomatal conductance, carbon assimilation, intrinsic water-use efficiency, transpiration and stem diameter volume growth (see statistical details in Table 2). The error bars represent mean ± standard error.

The area of new needles was larger in the offspring from control mother trees than in those from irrigated and irrigation-stop mother trees, regardless of drought and heat treatments (Table 2). Drought treatments reduced current-year needle area, irrespective of mother tree origins. However, we did not observe the effect of heating on the area of needles (Table 2 and Figure 2). Seedling-level stem diameter and volume growth were significantly lower among offspring from irrigated mother trees compared with those from control mother trees, irrespective of drought and heating treatments (Table 2 and Figure 2). The effects of drought and heat on volume growth were not significant (Table 2).

Significant differences in NSC concentrations (sugars and starch) were observed only between the irrigation and irrigation-stop mother tree treatments, across stems, bark and the whole plant, but not in roots or needles (Table 2). The drought treatment reduced NSC concentrations in new needles and coarse roots, whereas the heating treatment had no significant effects on any tree organ (Table 2 and Figure S3 available as Supplementary Data at *Tree Physiology* Online).

### Primary metabolites

The abundance of neophytadiene (involved in hormone regulation) and xylose (cell wall-related stress responses) were lower among offspring from irrigated mother trees compared with those from control mother trees (see Table S1 available as Supplementary Data at *Tree Physiology* Online). In contrast, phosphate (stress response regulation) and rhamnose (cell wall integrity) abundances were higher in offspring from irrigated mother trees (see Table S1 available as Supplementary Data at *Tree Physiology* Online). Moreover, the abundances of betaine and fructose (both involved in osmotic regulation) and xylose (involved in stress response) were lower in offspring from irrigation-stop mother trees compared with control mother trees. In contrast, the abundances of ethanolamine (no clear stress-related function), myristic acid and phosphate were higher (see Table S1 available as Supplementary Data at *Tree Physiology* Online). For the comparison between offspring from irrigation and irrigation-stop mother trees, betaine, chiro-inositol and ribonic acid 2 were lower in offspring from irrigation-stop mother trees. In contrast, ethanolamine and neophytadiene were higher (see Table S1 available as Supplementary Data at *Tree Physiology* Online). The drought treatment reduced the abundance of glycerol-glycoside (defense and oxidative stress response) while the heating treatment reduced the abundance of epigallocatechin (jasmonate pathway activation), galactinol (osmoprotection), gluconic acid (no clear stress related function), kaempferol (antioxidant), meso-erythritol (osmoprotection) and ononitol (osmoprotectant). Heating increased the abundance of 3-Hydroxypyridine, glycolic acid (redox balance), melezitose (no clear stress-related function) palmitic acid (membrane stabilization), tetradecane (jasmonate pathway regulation) and threonic acid (ascorbate metabolism and antioxidative response), regardless of mother tree origins (see Tables S1 and S2 available as Supplementary Data at *Tree Physiology* Online).

### Transcriptomic responses

#### General analysis of transcriptomic response in offspring

We found less correlation among the offspring of irrigated mother trees compared with the progenies from control or irrigation-stop mother trees (see Figure S4 available as Supplementary Data at *Tree Physiology* Online). A significantly higher percentage of genes were upregulated in offspring from irrigated mother trees compared with those from control mother trees, whereas gene expression was predominantly downregulated in offspring from irrigation-stop mother trees relative to the controls (Table 3 and Figures 3 and 4). Under hot-drought conditions, offspring from irrigation mother trees were associated with a downregulation of cell-killing processes, while under ambient temperature and well-watered conditions, they exhibited a downregulation of immune system processes and an upregulation of electron transfer activity (see Table S3 available as Supplementary Data at *Tree Physiology* Online).

**Table 3:** Genes and transcription factors involved in drought stress responses and tolerance differentially expressed in the progenies of the trees with diverse drought experience, growing under different environmental conditions. Note. C: control, IR: irrigation and IS: irrigation-stop, ↑: upregulated, ↓: downregulated.

Gene group or pathway	Number of analyzed genes (DEGs)	IR vs C	IR vs C under heat and drought	IR vs C under ambient temperature and water	IS vs C	IS vs C under heat and drought	IS vs C under ambient temperature and water
Ca^2+^-related proteins	195 (40)	6↑ 6↓	8↑5↓	6↑9↓	3↑12↓	3↑9↓	5↑17↓
CPK (calcium-dependent protein kinase)	19 (5)	1↑1↓	1↑1↓	1↑1↓	1↓	2↑2↓	1↑3↓
LEA	67 (26)	8↑2↓	7↑4↓	7↑	11↓	4↑7↓	2↑10↓
Dehydration-related + ERD (Early responsive to Dehydration)	78 (20)	3↑2↓	5↑1↓	8↑2↓	3↑	4↑2↓	2↑2↓
ABA/WDS induced protein (a family of plant proteins induced by water deficit stress (WDS), or abscisic acid (ABA) stress and ripening)	15 (11)	3↑1↓	1↑	3↑1↓	3↓	2↑1↓	2↓
NAC TF	51 (42)	14↑2↓	15↑2↓	13↑5↓	5↑15↓	4↑13↓	8↑15↓
MYC TF	3 (1)						1↓
MYB TF	72 (72)	14↑6↓	13↑9↓	14↑6↓	3↑25↓	6↑29↓	6↑ 18↓
DREB TF	10 (3)		1↑			2↑	1↓
bHLH TF	183 (37)	16↑5↓	7↑4↓	13↑3↓	3↑14↓	2↑9↓	3↑15↓
WRKY	114 (38)	11↑2↓	17↑2↓	8↑1↓	1↑21↓	2↑17↓	2↑22↓
							
Sum		76↑27↓	75↑28↓	73↑28↓	18↑102↓	31↑89↓	29↑106↓

Gene lists are presented in the Excel Table S1 available as Supplementary Data at *Tree Physiology* Online.

**Figure 3:**
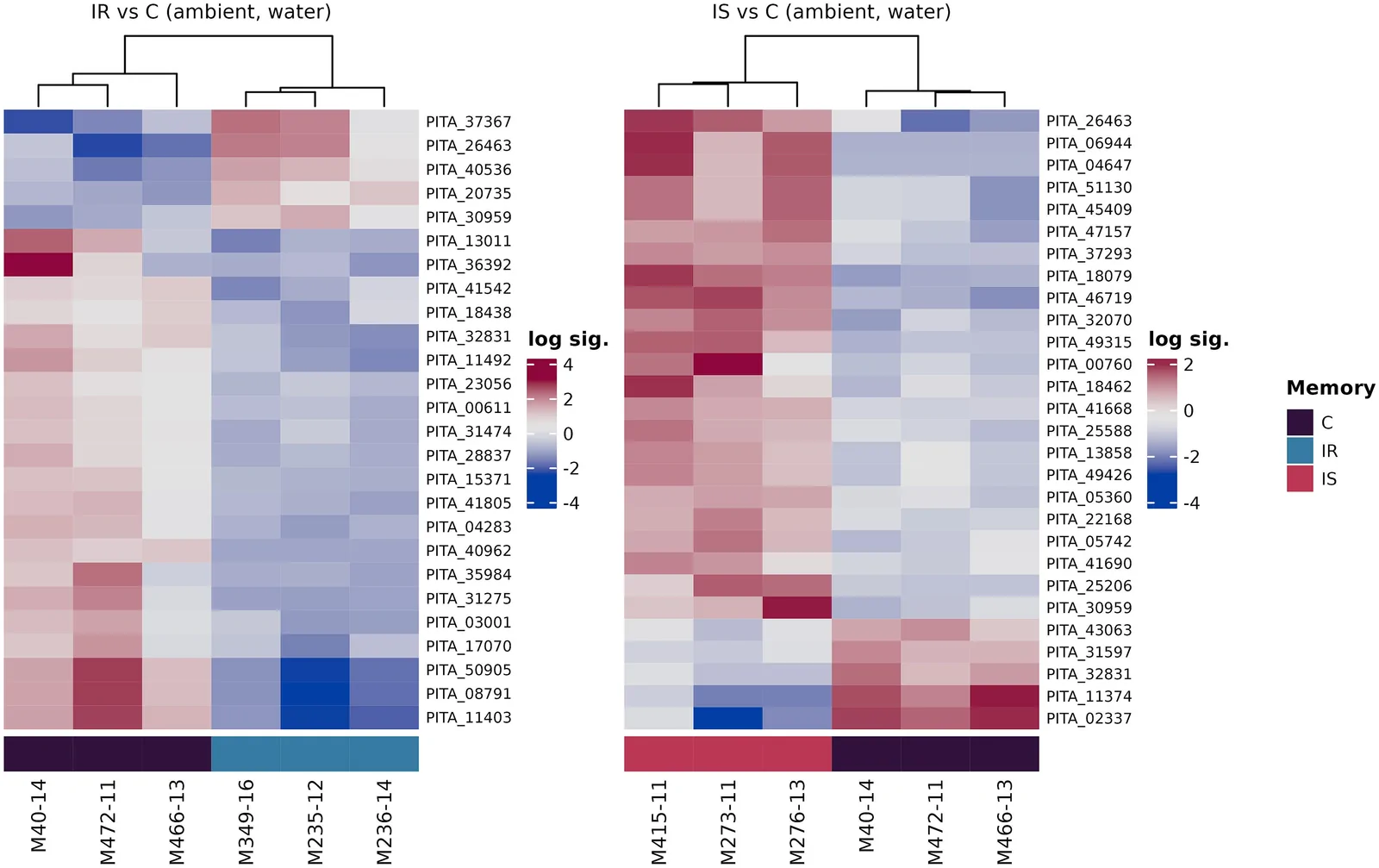
Heatmaps of differently expressed genes (*P* < 0.01) when offspring from irrigation vs control and irrigation-stop vs control mother trees grown under ambient heat and well-watered condition. Red gradient colors represent the upregulation while blue gradient colors represent the downregulation of gene functions. The memory C (control), IR (irrigation) and IS (irrigation-stop) represent offsprings from control, irrigation and irrigation-stop mother trees. Each column (e.g., M40–14) represents a sample.

**Figure 4:**
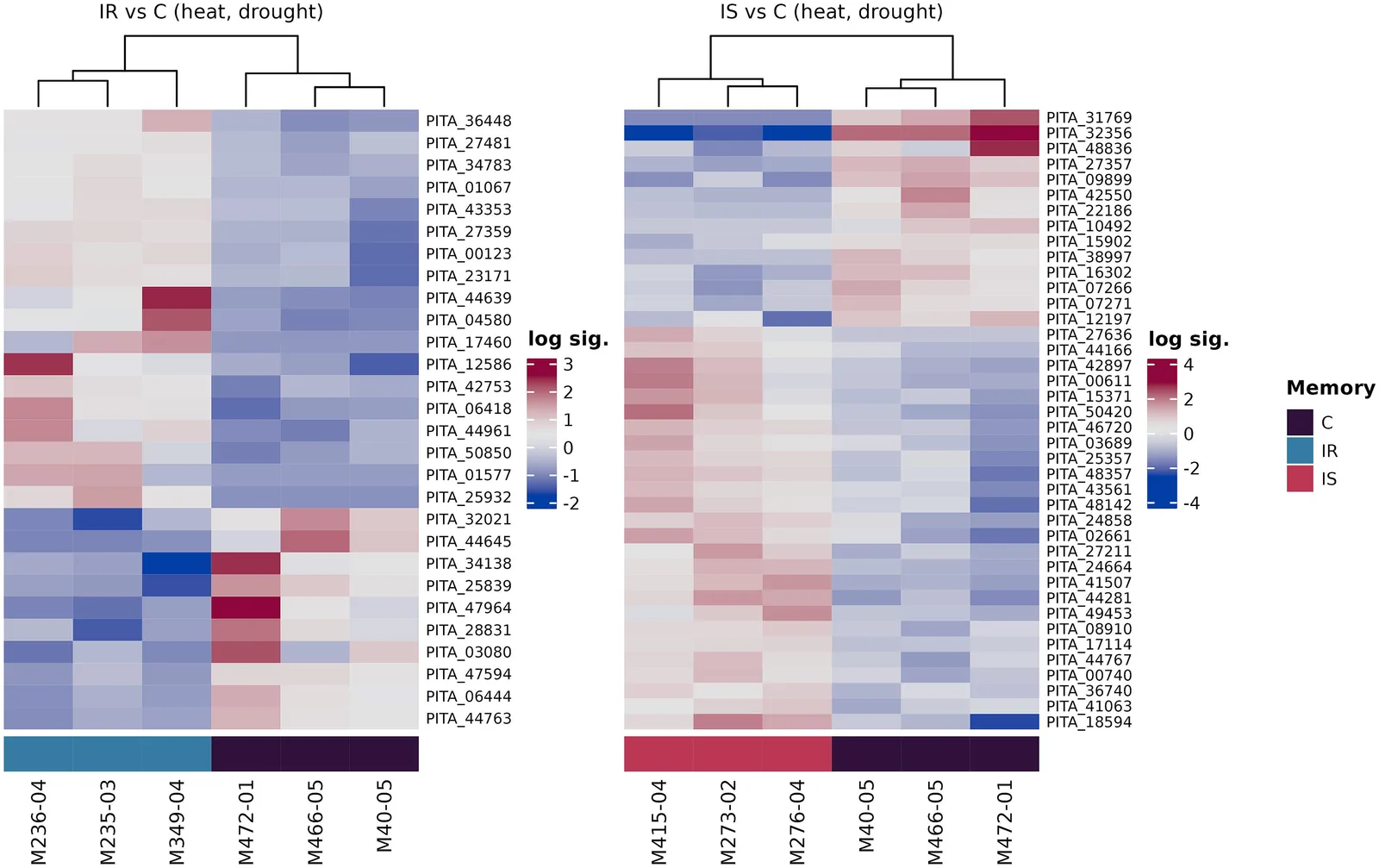
Heatmaps of differently expressed genes (*P* < 0.01) when offspring from irrigation vs control and irrigation-stop vs control grown under hot-drought (i.e., heat, drought) conditions. Red gradient colors represent the upregulation while blue gradient colors represent the downregulation of gene functions. The memory C (control), IR (irrigation) and IS (irrigation-stop) represent offsprings from control, irrigation and irrigation-stop mother trees. Each column (e.g., M236–04) represents a sample.

#### Genes and transcription factors involved in drought stress responses

We investigated the genes and transcription factors involved in drought stress responses, with a primary focus on the water deficit stress (WDS) and abscisic acid (ABA)-dependent pathways. Among 15 gene models of ABA/WDS proteins, 11 showed expression changes in the progenies under different irrigation conditions (Table 3 and Excel E1 and E2 available as Supplementary Data at *Tree Physiology* Online), with *PtASR1* (PITA 23545) notably displaying opposite regulation patterns between the treatments.

Of the 67 expressed late embryogenesis abundant (LEA) proteins, which include e.g., chaperons that also mediate stress responses, 26 were identified as differentially expressed genes across all seedling treatments (Table 3 and Excel E1 and E2 available as Supplementary Data at *Tree Physiology* Online). Eight genes were upregulated and two downregulated in irrigation progenies, while three specific genes showed opposite regulation between irrigation and irrigation-stop conditions (for details, see the extended results in the Table S4 and Excel E1 and E2 available as Supplementary Data at *Tree Physiology* Online). Dehydration-related genes generally maintain low expression levels across the treatments.

Analysis of Ca^2+^-related proteins contributing to stress tolerance and drought stress sensing revealed 40 differentially expressed genes out of 195 annotated proteins (Table 3 and Excel E1 and E2 available as Supplementary Data at *Tree Physiology* Online). Notable patterns included four transcripts (three calcium-binding proteins and one calcium-dependent protein kinase) showing upregulation in the progeny of irrigated trees and downregulation in that of irrigation-stop (for details, see the extended results in the Table S4 available as Supplementary Data at *Tree Physiology* Online).

Regarding transcription factors (TFs) involved in regulating signal transduction gene expression, out of 433 expressed TF genes, 193 showed differential expression (Table 3 and Excel E1 and E2 available as Supplementary Data at *Tree Physiology* Online). NAC, MYB, bHLH and WRKY TF are among the most differentially expressed. There were drastic differences in the TF gene expression. In the progenies from irrigated mother trees, the number of upregulated TF was always several times higher than that of downregulated ones for all comparisons with controls, drought or ambient conditions. Conversely, in the progeny of irrigation-stop mother trees, downregulated TF genes predominated in all comparisons (Table 3).

#### Epigenetic mechanisms-related genes

Analysis of genes conveying epigenetic mechanisms revealed differential expression patterns for some genes involved in the epigenetic memory machinery. Regarding DNA methylation, we defined 17 gene models containing the C5 cytosine-specific DNA methylase domain, including six similar to MET2 and DNMT2, seven to CMT (Chromomethylase) genes and three to DRMs (Domains Rearranged Methyltransferase) genes. Only seven showed treatment-responsive differential expression, with only two upregulated under heat and drought conditions, and all others were downregulated (Table 4).

**Table 4:** Genes involved in the epigenetic mechanism pathways differentially expressed during drought stress response in the progenies of the trees with diverse drought experience, growing in the different environmental conditions.

Gene group or pathway	Number of analyzed genes (DEGs)	Irrigation vs control		Irrigation vs control under heat and drought		Irrigation vs control under ambient temperature and water		Irrigation-stop vs control		Irrigation-stop vs control under heat and drought		Irrigation-stop vs control under ambient temperature and water	
		Up	Down	Up	Down	Up	Down	Up	Down	Up	Down	Up	Down
DNA methylases	17 (7)			1	1		2		2	1	2	1	2
Histone methylation	113 (11)		1	1	1	1	3		1		3	1	1
HATs	49 (8)	1		3		2	1	1	4	1	2	1	2
HDACs	31(4)					1	1		1		2	1	1
C^me^ readers (MBD proteins)	37 (9)	1	2	1			2	2	1	1	1		
Chromatin remodeling	125 (28)	2	2	3	2	3	13	1	3	2	4		3
AGO	41 (6)		2	1			1			1	1		1
Dicer	42 (6)	1	1		1	1	3	1		1	2		
sRNA pathway	33 (3)						2	1	1	1			1
RdDM pathway	144 (35)	5	3	11	1	3	10	5	3	5	7	5	5

Gene lists are presented in the Excel Table S2 available as Supplementary Data at *Tree Physiology* Online.

Among the 113 histone methyltransferase genes, 11 were differentially expressed genes (DEGs) (Table 4), with two stably upregulated genes for irrigation and one downregulated for irrigation-stop progenies. Of the 49-histone acetyltransferase (HAT) genes, 8 were DEGs, with four showing upregulation in the irrigation progeny. Of the 31 HDAC complex genes, only four showed differential expression. Among the 125 chromatin remodelers, 28 were DEGs, with eight genes inconsistently upregulated in irrigation and five in irrigation-stop offspring (for details, see the extended results in the Table S4 available as Supplementary Data at *Tree Physiology* Online).

For epigenetic readers, 37 MBD (Methyl-CpG-binding domain) genes were identified, of which 10 were DEGs. Of the 102 PHD finger protein genes, 12 showed differential expression, including one upregulated in the irrigation progeny under heat stress. 59 Chromo motif-containing genes (7 DEGs) and 48 bromodomain-containing protein genes (6 DEGs) were identified. Notable findings included 450+ WD40-containing proteins, with 29 upregulated in irrigation and 12 upregulated in irrigation-stop offspring, mostly under heat stress (for details, see the extended results in the Table S4 available as Supplementary Data at *Tree Physiology* Online).

For small RNA processing pathway genes, the analysis of 42 DICER-like genes showed six differential expression genes. Most of them were downregulated in stressful conditions, except one, upregulated in both irrigation and irrigation-stop progenies. Similarly, among the 41 ARGONAUTE-like genes, six were DEGs, with three showing upregulation under specific conditions. Among the 144 RNA-directed DNA methylation pathway genes (RdDM), 35 were DEGs. The three RDRs showed differential regulation, with upregulation patterns in both irrigation and irrigation-stop offspring under heat stress. Additional components included 25 SHH-like transcripts (one upregulated in the irrigation progeny), 70 POL-like transcripts (20 DEGs) and 33 XH/XS domain transcripts (seven upregulated in irrigation and two in both irrigation and irrigation-stop offspring) (for details, see the extended results in the Table S4 available as Supplementary Data at *Tree Physiology* Online).

## Discussion

Offspring derived from drought-exposed mother trees exhibited higher carbon assimilation, but not WUEi or increased growth, even under irrigated conditions. This pattern contrasts with the findings of Bose et al. (2020). In that previous experiment, offspring from irrigated mother trees in the year of germination performed best under irrigated conditions, whereas offspring from drought-experienced mother trees showed enhanced growth and survival under hot-drought conditions, indicating environment-specific transgenerational memory effects (Walter et al. 2016). While these responses suggested transgenerational environmental memory, the relative contributions of maternal provisioning versus epigenetic mechanisms could not be fully resolved. Notably, mother trees did not differ in genotype, and seed mass did not vary between treatments, indicating a limited role of maternal effects. This previous assumption is corroborated by our N and δ^15^N analyses, which show comparable maternal nutrient provisioning.

In the present study, offspring originating from drought-exposed mother trees consistently outperformed those from irrigation-experienced mother trees, regardless of the applied drought or irrigation treatments. Specifically, these offspring exhibited significantly higher carbon assimilation, while WUEi did not differ across treatments. The increased carbon assimilation was accompanied by greater needle area, larger stem diameter and volume growth, indicating improved overall physiological performance and carbon gain rather than increased water-use efficiency (Figure 2 and Table 2). The consistently strong performance of drought-lineage offspring across contrasting environmental conditions suggests a broad enhancement of resilience and acclimation capacity, potentially driven by inherited acclimation or memory effects shaped by the drought experience of the maternal trees (Sow et al. 2021).

The weaker performance observed in offspring derived from irrigated mother trees (compared with the drought lineage) may be attributed to the 3-year period of exposure to non-treated conditions prior to their re-exposure to experimental irrigation. This extended exposure to environmental conditions which differed from those experienced by the maternal trees likely resulted in a mismatch between inherited patterns of trait plasticity and the conditions encountered during early development (Hilker and Schmülling 2019, Mayer and Charron 2021). Offspring from irrigated mother trees are hypothesized to exhibit epigenetically mediated physiological responses and plasticity patterns optimized for high water availability (Walter et al. 2016, Bose et al. 2020). However, maintenance under near-ambient (non-irrigated) conditions—where irrigation was applied only to prevent mortality—may have altered or dampened these inherited plastic responses. Prolonged exposure to contrasting conditions may thus weaken or modify the parental ‘irrigation memory’, constraining the offspring’s ability to respond effectively to subsequent irrigation (under which they initially performed well) as well as to drought stress (Crisp et al. 2016). This pattern highlights the potential long-term consequences of environmentally induced memory on offspring performance and adaptability (Lämke and Bäurle 2017). While parental irrigation may prime offspring for enhanced performance under high water availability, such environmentally tuned plasticity can cause maladjustments when conditions shift to more variable environments (Mayer and Charron 2021). The apparent loss or mismatch of inherited plasticity underscores the dynamic and context-dependent nature of environmental memory (Lämke and Bäurle 2017) and its role in shaping growth and physiological responses of tree offspring under changing conditions.

Further investigation is needed to disentangle the complex interactions among parental (intra-generational) response patterns and plasticity, their transgenerational transmission to offspring and shifts in environmental variability between generations—processes that are particularly important in long-lived species such as trees (Raj et al. 2011, Bräutigam et al. 2013, Ramírez-Valiente et al. 2021). In the present study, our analyses were limited to transcriptomic and metabolomic responses, which capture only two layers of the complex regulatory processes underlying stress memory and phenotypic variation. Although transcriptional and metabolic changes provide important insights into physiological regulation, they may not fully explain the persistence or erosion of intergenerational memory effects. Epigenetic mechanisms such as DNA methylation, histone modifications and small RNA–mediated regulation have been implicated in the establishment, maintenance and transmission of stress memory in plants (Yakovlev et al. 2010, Sow et al. 2021, Panda et al. 2023). Even though we focused our transcription analyses on the expression of genes involved in epigenetic processes (methylation, epigenetic readers) and small RNA processing pathway, the lack of direct small RNA and methylation profiles limits our ability to link maternal environmental history to stable heritable modifications. Furthermore, although our focus on the 3-year endpoint allowed us to assess whether maternal effects persisted after prolonged exposure to non-treated conditions, the absence of intermediate measurements during the first and second years prevents us from reconstructing the temporal trajectory of memory persistence or loss. Consequently, we cannot determine whether the observed effects were gradually attenuated over time, remained relatively stable before declining, or were lost earlier during offspring development. Integrative approaches combining transcriptomics with epigenomic and small RNA profiling, ideally across multiple developmental stages and environmental contexts, will be critical for advancing our understanding of how stress experiences are encoded, retained or reset across generations in forest trees. In addition to those limitations, relative humidity and photosynthetic photon flux density at canopy height were not monitored during the experiment, and vapor pressure deficit was not possible to calculate. The absence of these environmental measurements limits a complete characterization of the chamber microclimate and should be considered when interpreting the warming and gas-exchange responses.

Although the drought treatment reduced mean soil VWC by ~51%, its effects on stem diameter and volume growth were relatively small (Table 2). This apparent discrepancy may reflect both the moderate, intermittent nature of the imposed drought and physiological adjustment by the seedlings. Drought-treated seedlings received limited water periodically, resulting in repeated drying and partial-rewetting cycles rather than continuous soil drying. The ~10% reductions in stomatal conductance and transpiration indicate that seedlings restricted water loss in response to reduced soil water availability. Such regulation may have helped maintain plant water status and structural growth. However, whole-plant NSC concentration and pool size declined by ~12% and 26%, respectively, suggesting that maintaining growth under drought may have come partly at the expense of stored carbon reserves.

Heat and drought stress are strong environmental factors affecting plant growth and productivity (Arend et al. 2011, Perrella et al. 2022). Plants have evolved complex mechanisms to perceive, respond to and cope with heat and drought stress through diverse strategies, including tolerance, avoidance and escape (Bréda et al. 2006, Blanco-Sánchez et al. 2022, Elfarargi et al. 2026). These mechanisms involve the activation of specific genes and transcription factors (TFs) that regulate physiological, biochemical and molecular responses (Raj et al. 2011, Perrella et al. 2022). The phytohormone abscisic acid (ABA) plays a pivotal role in the complex mechanisms plants employ in response to drought stress, including regulating stomatal closure and activating drought-responsive genes (Movahedi et al. 2023). Abscisic acid is critical for mediating hormonal and osmotic signaling pathways during drought conditions, facilitating three primary drought-coping strategies: escaping drought by adjusting developmental timing, avoiding dehydration through physiological adjustments and tolerating desiccation via biochemical adaptations (Fang and Xiong 2015, Cao et al. 2017). Our findings revealed that 11 out of 15 analyzed ABA-related genes exhibited changes in expression in progeny of mother trees subjected to different irrigation regimes (Table 3). Additionally, stress tolerance proteins, particularly late embryogenesis abundant (LEA) proteins, showed differential expression patterns. Among the 67 LEA-related genes examined, 26 displayed significant expression changes, with only three genes consistently upregulated in progeny from irrigated mothers (IR) and downregulated in progeny from irrigation-stop mothers (IS). Notably, a smaller subset of dehydration-related genes demonstrated differential expression across treatments, suggesting a limited and inconsistent response in these pathways.

The integration of primary metabolite and transcriptomic analyses reveals a coordinated yet modest response to maternal irrigation history and seedling stress treatments, with some implications for understanding transgenerational mechanisms. Both datasets demonstrate that offspring from irrigated mother trees exhibit slightly distinct molecular profiles compared with controls, with metabolite changes (e.g., elevated osmoprotectants like trehalose and stress signaling compounds like myristic acid) paralleling differential expression of stress-responsive gene families, including LEA proteins, ABA/WDS pathway genes and transcription factors (particularly NAC (NAM, ATAF1/2 and CUC2), MYB (Myeloblastosis) and WRKY (WRKYGQK amino acid) families). When seedling treatments are considered, the heating treatment elicited increasing abundances of membrane stabilizers (palmitic acid) and antioxidative response metabolites (threonic acid) that may align with the observed downregulation of cell-killing processes in gene expression profiles under hot-drought conditions.

Among signaling factors, we analyzed Ca^2+^-related proteins. Stretch-activated calcium channels are thought to function as osmosensors in drought stress sensing (Shinozaki and Yamaguchi-Shinozaki 2022). The functional role of Ca^2+^ is very well known in signaling the emergence of stress and then eliciting the activation of an appropriate tolerance mechanism (Sharma and Kumar 2021). The concentration of free cytosolic Ca^2+^ transiently increases in response to various abiotic stresses such as cold, drought and high salinity. Active transport of Ca^2+^ from the cytosol into the apoplast or to the intracellular organelles is driven by Ca^2+^ ATPases (PMCA) and H^+^/Ca^2+^ antiporters (CAX). The increase in the concentration of Ca^2+^ is sensed by some calcium-binding proteins (CBPs) or sensor proteins, which lead to the activation of different calcium-dependent protein kinases (CPK). These kinases play an important role in regulating the function of a myriad of genes including stress-responsive genes, resulting in stress tolerance. It was unexpected that only a few genes were differentially expressed between progenies of different treatments.

Similarly, from the large number of genes related to epigenetic mechanisms, only a few were differentially expressed in progenies of mother trees with different irrigation treatments (Table 4). These lack of differences, in general, confirm the lack or low level of epigenetic differences in progenies after long-term growth in natural conditions. This relatively small number of DEGs is also reflected in the mostly minor differences in primary metabolites among progenies. This suggests that transgenerational memory operates through complex regulatory mechanisms beyond simple transcriptional control. Assessments of old-growth Scots pine indicated that over large gradients of stress and loss of vitality, the primary needle metabolome remains homeostatic (Hunziker et al. 2024). Significant changes in the metabolome were observed only when irreversible structural damage leading to mortality occurred. Since the primary metabolisms maintain the essential cellular functions and provide precursors for many specialized metabolites, homeostasis of metabolite availability (indicated by the metabolite abundance as assessed in our study) is critical (Shulaev et al. 2008). Our results still show that various stress-related compounds changed their abundance in response to the maternal history and seedling treatment. However, a lack of change in the abundance of other metabolites does not preclude changes in flux and metabolic network regulation. Given the relatively low number of differentially regulated genes among the different progenies, such regulatory processes (if active at all) should thus act on a post-transcriptional level (Grüning et al. 2010).

Progeny from different maternal irrigation regimes exhibited differential gene expression; however, the number of significantly altered genes was relatively small, and expression patterns varied across functional categories. At the metabolite level, differences among progenies were likewise minor, consistent with the gene expression patterns. In contrast, clear differences in phenotypic traits and gas-exchange physiology were observed among offspring from mother trees exposed to different treatments. This contrasts with the initial hypothesis, which anticipated weaker phenotypic divergence following the 3-year non-treatment period, alongside stronger differences at the metabolomic and transcriptomic levels. These patterns may suggest that epigenetic effects and transgenerational memory remain active, but that the links between differential gene expression, primary metabolism and drought-related phenotypic responses likely involve additional regulatory layers or environmental interactions. This highlights the complexity of drought acclimation mechanisms in tree offspring.

Overall, our findings emphasize the intricate interplay between parental environmental memory, gene expression and developmental environmental conditions. Transgenerational memory of environmental conditions may help young seedlings in their initial establishment phase to better adjust to the parental environment (Bose et al. 2020). This potential, however, may fade over time and even turn into maladjustment depending on the combination of conditions experienced by the parents and the offspring (this study). Our results highlight the importance of further studying these interactions and interferences to better understand how traits and functions in tree offspring are shaped and expressed under changing environmental conditions.

## Supplementary Material

Supplementary_material_tpag141

## Data Availability

The data supporting the findings of this study are available from the corresponding author upon request.
