# Supplementary material for: When environments mismatch—the limits of transgenerational climate memory in Scots pine
Source: Tree Physiol. 2026 Sep 23;46(10):tpag141. doi: 10.1093/treephys/tpag141 (PMC13685802; doi:10.1093/treephys/tpag141)
Supplement: Supplementary file 1 [file supplementary_material_tpag141.zip › Supplementary materials.docx]

**Supplementary Materials to « When environments mismatch - The limits of transgenerational climate memory in Scots pine »**

by Na Luo, Arthur Gessler, Falko Noé, Igor A. Yakovlev, Isabel Weirich, Bernd Kammerer, Rie Shimizu-Inatsugi, Stefan Hunziker, Simon Lagies, Simone Maria Pieber, Daniel Mohl, Ugo Molteni, Kentaro Shimizu, and Arun K. Bose

The document contains three types of Supplementary Materials:

- Supplementary Table (S1 to S4)
- Supplementary Figure S1
- Two additional supplementary Excel files (E1 to E2) are as separate files

**Supplementary Table S1.** Results of the statistical analysis for all primary metabolites included in the study, based on linear mixed-effects models. Values are presented as coefficients ± standard errors with their associated *p*-values. Bold values indicate statistically significant differences (*p* < 0.05). Pairwise comparisons and corresponding *p*-values were obtained using post-hoc Tukey multiple comparison tests.

| Primary metabolites | Mother tree treatment | | | Drought  *vs*  watered | Ambient heat  *vs*  ambient + 4 °C |
| --- | --- | --- | --- | --- | --- |
|  | Control  *vs*  Irrigation | Control  *vs*  Irrigation-stop | Irrigation *vs* Irrigation-stop |  |  |
| 4.6-dimethyl- Dodecane | -0.004±0.004  (0.632) | -0.004±0.004  (0.533) | -0.001±0.004  (0.986) | -0.002±0.003  (0.512) | 0.001±0.003  (0.702) |
| 1.6-Anhydro-D-Glucose | 0.003±0.010  (0.954) | -0.001±0.010  (0.993) | -0.004±0.010  (0.915) | -0.013±0.008  (0.111) | -0.001±0.008  (0.909) |
| 2-Hydroxypyridine | 0.004±0.014  (0.952) | 0.005±0.014  (0.926) | 0.001±0.014  (0.997) | -0.011±0.012  (0.339) | 0.005±0.012  (0.623) |
| 3-Hydroxypyridine | 0.034±0.017  (0.111) | 0.010±0.017  (0.814) | -0.024±0.017  (0.333) | -0.012±0.014  (0.383) | **0.030±0.014**  **(0.030)** |
| 3.6-dimethyl- Decane | 0.001±0.004  (0.962) | 0.008±0.004  (0.165) | 0.006±0.004  (0.265) | 0.001±0.003  (0.781) | 0.004±0.003  (0.301) |
| 4-Coumaric acid | -0.043±0.055  (0.712) | -0.073±0.055  (0.383) | -0.030±0.055  (0.850) | 0.011±0.045  (0.799) | -0.017±0.045  (0.712) |
| 4-Hydroxybenzoic acid | 0.001±0.005  (0.974) | 0.003±0.005  (0.824) | 0.002±0.005  (0.925) | -0.004±0.004  (0.286) | -0.003±0.004  (0.432) |
| Abietic acid | 0.013±0.065  (0.978) | 0.062±0.065  (0.605) | 0.049±0.065  (0.729) | -0.084±0.053  (0.116) | -0.029±0.053  (0.589) |
| Alpha-Tocopherol | -0.031±0.018  (0.211) | -0.037±0.018  (0.105) | -0.006±0.018  (0.932) | -0.018±0.015  (0.233) | -0.021±0.015  (0.158) |
| Aspartic acid | 0.050±0.041  (0.439) | -0.029±0.041  (0.754) | -0.080±0.041  (0.133) | -0.011±0.033  (0.748) | 0.054±0.033  (0.112) |
| Benzoic acid | 0.006±0.027  (0.971) | 0.035±0.027  (0.393) | 0.029±0.027  (0.528) | -0.001±0.022  (0.956) | 0.005±0.022  (0.829) |
| Beta-Sitosterol | 0.111±0.108  (0.562) | 0.124±0.108  (0.491) | 0.013±0.108  (0.993) | -0.090±0.089  (0.314) | 0.040±0.089  (0.655) |
| Betaine | 0.582±0.438  (0.383) | **1.945±0.438**  **(0.000)** | **1.362±0.438**  **(0.008)** | -0.532±0.357  (0.141) | 0.151±0.357  (0.674) |
| Caprylic acid | -0.006±0.010  (0.807) | 0.005±0.010  (0.854) | 0.011±0.010  (0.480) | -0.010±0.008  (0.231) | -0.001±0.008  (0.939) |
| Catechine (2R-E) | -0.409±0.398  (0.561) | -0.198±0.398  (0.873) | 0.212±0.398  (0.856) | -0.528±0.325  (0.109) | -0.609±0.325  (0.065) |
| Catechine 1 | -0.001±0.005  (0.974) | 0.005±0.005  (0.483) | 0.006±0.005  (0.360) | -0.001±0.004  (0.863) | -0.001±0.004  (0.798) |
| Chiro-Inositol | -0.073±0.059  (0.438) | 0.075±0.059  (0.420) | **0.147±0.059**  **(0.039)** | -0.074±0.048  (0.130) | 0.091±0.048  (0.062) |
| Citric acid | -0.969±0.593  (0.239) | -0.027±0.593  (0.999) | 0.942±0.593  (0.258) | -0.598±0.485  (0.221) | 0.649±0.485  (0.185) |
| Decane | 0.003±0.009  (0.951) | -0.005±0.009  (0.863) | -0.008±0.009  (0.692) | 0.004±0.008  (0.560) | 0.007±0.008  (0.363) |
| Dehydroabietic acid/  Callitrisic acid | -0.225±0.114  (0.125) | 0.007±0.114  (0.998) | 0.232±0.114  (0.110) | -0.145±0.093  (0.124) | 0.094±0.093  (0.314) |
| Dehydroascorbic acid | 0.015±0.126  (0.993) | 0.064±0.126  (0.868) | 0.049±0.126  (0.919) | -0.131±0.103  (0.205) | -0.107±0.103  (0.301) |
| Docosanol | -0.018±0.009  (0.144) | -0.001±0.009  (0.997) | 0.017±0.009  (0.166) | -0.011±0.008  (0.170) | -0.005±0.008  (0.494) |
| Dodecane | -0.002±0.005  (0.912) | 0.004±0.005  (0.653) | 0.006±0.005  (0.404) | -0.004±0.004  (0.262) | 0.0001±0.004  (0.974) |
| Dodecanoic acid | -0.006±0.005  (0.524) | -0.007±0.005  (0.341) | -0.002±0.005  (0.945) | -0.002±0.004  (0.673) | -0.006±0.004  (0.159) |
| Epigallocatechin | -0.212±0.230  (0.628) | 0.199±0.230  (0.665) | 0.410±0.230  (0.182) | -0.011±0.188  (0.954) | **-0.629±0.188**  **(0.001)** |
| Erythronolactone | 0.011±0.011  (0.555) | -0.003±0.011  (0.965) | -0.014±0.011  (0.403) | 0.008±0.009  (0.380) | -0.002±0.009  (0.795) |
| Ethanolamine | 0.015±0.017  (0.654) | **-0.045±0.017**  **(0.028)** | **-0.060±0.017**  **(0.002)** | 0.012±0.014  (0.394) | 0.004±0.014  (0.764) |
| Ferulic acid | 0.004±0.005  (0.689) | -0.002±0.005  (0.927) | -0.006±0.005  (0.460) | -0.004±0.004  (0.330) | -0.002±0.004  (0.634) |
| Fructose | 13.715±10.391  (0.389) | **25.889±10.391**  **(0.040)** | 12.17±10.391  (0.474) | -11.343±8.485  (0.186) | 9.211±8.485  (0.282) |
| Galactaric acid | 0.040±0.021  (0.131) | 0.028±0.021  (0.368) | -0.012±0.021  (0.822) | 0.001±0.017  (0.950) | -0.009±0.017  (0.593) |
| Galactinol | 0.102±0.135  (0.732) | 0.069±0.135  (0.865) | -0.032±0.135  (0.969) | 0.120±0.110  (0.282) | **-0.522±0.110**  **(0.000)** |
| Gentiobiose | 0.043±0.033  (0.385) | 0.015±0.033  (0.890) | -0.028±0.033  (0.663) | -0.036±0.027  (0.184) | 0.030±0.027  (0.265) |
| Gluconic acid | -0.875±0.457  (0.143) | 0.096±0.457  (0.976) | 0.970±0.457  (0.093) | -0.238±0.373  (0.525) | **-0.799±0.373**  **(0.036)** |
| Glucose | 17.890±14.417  (0.434) | 31.72±14.417  (0.078) | 13.838±14.417  (0.605) | -16.639±11.77  (0.162) | 9.077±11.771  (0.443) |
| Glutamic acid | 0.211±0.124  (0.210) | 0.019±0.124  (0.988) | -0.193±0.124  (0.271) | -0.055±0.066  (0.407) | 0.058±0.066  (0.380) |
| Glutamic acid  (dehydrated) | 0.096±0.081  (0.464) | -0.026±0.081  (0.946) | -0.122±0.081  (0.294) | -0.007±0.101  (0.942) | 0.200±0.101  (0.051) |
| Glyceric acid | 0.034±0.027  (0.421) | -0.030±0.027  (0.509) | -0.064±0.027  (0.053) | -0.016±0.022  (0.472) | -0.018±0.022  (0.420) |
| Glycerol-glycoside | -0.061±0.111  (0.847) | 0.026±0.111  (0.970) | 0.087±0.111  (0.713) | **-0.232±0.091**  **(0.013)** | -0.068±0.091  (0.455) |
| Glycerol | 0.031±2.160  (1.000) | 1.883±2.160  (0.660) | 1.852±2.160  (0.669) | -3.328±1.764  (0.071) | -2.721±1.764  (0.128) |
| Glycerol 3-phosphate | 0.005±0.005  (0.636) | -0.002±0.005  (0.947) | -0.007±0.005  (0.442) | 0.001±0.004  (0.839) | -0.005±0.004  (0.250) |
| Glycine | 0.011±0.008  (0.348) | 0.011±0.008  (0.391) | -0.001±0.008  (0.996) | 0.001±0.007  (0.902) | 0.011±0.007  (0.095) |
| Glycolic acid | 0.0004±0.005  (0.997) | 0.006±0.005  (0.426) | 0.006±0.005  (0.471) | -0.002±0.004  (0.618) | **0.018±0.004**  **(0.000)** |
| Hexanoic acid | -0.012±0.014  (0.660) | 0.007±0.014  (0.879) | 0.018±0.014  (0.369) | -0.001±0.011  (0.942) | -0.004±0.011  (0.688) |
| Hydroxylamine | 0.006±0.014  (0.915) | -0.007±0.014  (0.856) | -0.013±0.014  (0.620) | -0.004±0.011  (0.749) | 0.007±0.011  (0.517) |
| Kaempferol | -0.002±0.002  (0.529) | -0.002±0.002  (0.659) | 0.001±0.002  (0.976) | -0.003±0.002  (0.072) | **-0.003±0.002**  **(0.041)** |
| Lactic acid | 0.014±0.060  (0.968) | 0.023±0.060  (0.920) | 0.009±0.060  (0.988) | -0.017±0.049  (0.734) | -0.035±0.049  (0.476) |
| Malic acid | -0.494±0.439  (0.501) | -0.208±0.439  (0.884) | 0.287±0.439  (0.791) | 0.225±0.358  (0.532) | 0.689±0.358  (0.059) |
| Maltitol | -0.473±0.250  (0.148) | -0.001±0.250  (1.000) | 0.472±0.250  (0.150) | -0.363±0.204  (0.080) | -0.075±0.204  (0.714) |
| Mannitol | -0.135±0.246  (0.848) | -0.045±0.246  (0.982) | 0.090±0.246  (0.929) | -0.392±0.201  (0.055) | -0.051±0.201  (0.802) |
| Melezitose | -2.391±6.622  (0.931) | 0.051±6.622  (1.000) | 2.442±6.622  (0.928) | -7.309±5.407  (0.181) | **11.642±5.407**  **(0.035)** |
| Melibiose | -0.018±0.067  (0.961) | -0.030±0.067  (0.896) | -0.012±0.067  (0.983) | -0.081±0.055  (0.143) | 0.066±0.055  (0.237) |
| Meso-Erythritol | -0.017±0.129  (0.991) | -0.131±0.129  (0.568) | -0.114±0.129  (0.649) | -0.165±0.105  (0.120) | **-0.263±0.105**  **(0.015)** |
| Myo-Inositol | 5.148±3.325  (0.275) | -2.473±3.325  (0.738) | -7.621±3.325  (0.064) | -0.831±2.715  (0.760) | -4.248±2.715  (0.122) |
| Myristic acid | -0.040±0.017  (0.068) | **-0.047±0.017**  **(0.024)** | -0.008±0.017  (0.902) | -0.011±0.014  (0.451) | 0.008±0.014  (0.586) |
| Neophytadiene | **0.146±0.045**  **(0.005)** | 0.006±0.045  (0.990) | **-0.140±0.045**  **(0.008)** | -0.005±0.037  (0.899) | 0.007±0.037  (0.848) |
| Oleic acid | -0.004±0.016  (0.958) | 0.000±0.016  (1.000) | 0.004±0.016  (0.963) | -0.011±0.013  (0.387) | 0.012±0.013  (0.368) |
| Ononitol | 2.626±3.272  (0.703) | 1.122±3.272  (0.937) | -1.504±3.272  (0.890) | 0.961±2.672  (0.720) | **-6.006±2.672**  **(0.028)** |
| Oxalic acid | -0.046±0.138  (0.940) | 0.100±0.138  (0.747) | 0.147±0.138  (0.539) | 0.086±0.113  (0.449) | -0.094±0.113  (0.408) |
| Palmitic acid | -1.352±0.826  (0.238) | -1.476±0.826  (0.182) | -0.124±0.826  (0.988) | -0.660±0.675  (0.331) | **1.611±0.675**  **(0.020)** |
| Phosphate | **-9.518±3.014**  **(0.007)** | **-8.605±3.014**  **(0.016)** | 0.914±3.014  (0.951) | -0.465±2.461  (0.851) | 4.196±2.461  (0.093) |
| Phytol | 0.032±0.020  (0.256) | -0.007±0.020  (0.934) | -0.039±0.020  (0.134) | 0.017±0.016  (0.288) | -0.010±0.016  (0.530) |
| Pinitol | -1.912±18.059  (0.994) | 26.94±18.059  (0.301) | 28.85±18.059  (0.254) | -20.680±14.74  (0.165) | -5.520±14.74  (0.709) |
| Quinic acid | -6.202±16.116  (0.922) | 7.899±16.116  (0.876) | 14.101±16.116  (0.658) | 18.575±13.158  (0.163) | -2.876±13.15  (0.828) |
| Raffinose | -0.112±0.572  (0.979) | 0.086±0.572  (0.988) | 0.198±0.572  (0.936) | 0.374±0.467  (0.426) | -0.535±0.467  (0.257) |
| Rhamnose | **-0.033±0.013**  **(0.040)** | -0.018±0.013  (0.395) | 0.016±0.013  (0.470) | 0.004±0.011  (0.698) | 0.007±0.011  (0.530) |
| Ribonic acid 1 | -0.103±0.046  (0.071) | -0.018±0.046  (0.920) | 0.085±0.046  (0.159) | 0.014±0.037  (0.707) | -0.017±0.037  (0.646) |
| Ribonic acid 2 | -0.044±0.027  (0.248) | 0.048±0.027  (0.196) | **0.091±0.027**  **(0.004)** | 0.016±0.022  (0.482) | -0.026±0.022  (0.244) |
| Shikimic acid | -0.025±17.827  (1.000) | 1.664±17.827  (0.995) | 1.689±17.827  (0.995) | -8.314±14.555  (0.570) | -6.712±14.55  (0.646) |
| Stearic acid | -1.145±0.873  (0.394) | -1.188±0.873  (0.367) | -0.042±0.873  (0.999) | -0.586±0.713  (0.414) | 1.321±0.713  (0.068) |
| Succinic acid | 0.021±0.019  (0.450) | 0.011±0.019  (0.827) | -0.010±0.019  (0.850) | -0.010±0.015  (0.517) | 0.008±0.015  (0.589) |
| Sucrose | -6.708±11.469  (0.829) | 8.604±11.469  (0.735) | 15.31±11.469  (0.381) | -5.703±9.364  (0.545) | 2.156±9.364  (0.819) |
| Tetradecane | 0.0001±0.007  (0.999) | 0.005±0.007  (0.778) | 0.005±0.007  (0.786) | -0.007±0.006  (0.235) | **0.012±0.006**  **(0.039)** |
| Threonic acid | -0.025±0.029  (0.676) | -0.0003±0.029  (0.999) | 0.025±0.029  (0.683) | 0.014±0.024  (0.563) | **0.051±0.024**  **(0.036)** |
| Trehalose | -0.209±0.094  (0.074) | -0.072±0.094  (0.727) | 0.137±0.094  (0.314) | -0.036±0.077  (0.636) | 0.052±0.077  (0.496) |
| Xylose | **0.418±0.128**  **(0.005)** | **0.376±0.128**  **(0.013)** | -0.041±0.128  (0.944) | -0.155±0.105  (0.145) | -0.059±0.105  (0.575) |

**Table S2**. Metabolites that significantly changed in abundance among offsprings of mother trees or seedling treatments. The stress-related mechanisms they are known to influence, along with the plant species studied, are listed below.

- **Neophytadiene**
  - Stress response function: Volatile derived from chlorophyll degradation during Tomato chlorosis virus infection; attracts whiteflies via BtOBP2 and mediates virus–vector–plant interactions. (Shi *et al.*, 2025)
  - Species: Tomato (*Solanum lycopersicum*), *Bemisia tabaci*
- **Xylose**
  - Stress response function: Integrity of the xylan backbone modulates drought responses; rice β1,2‑xylosyltransferase (RCN11) is required for abiotic stress responses; xylo‑oligosaccharides can act as cell wall–derived signals in defense contexts (Takano *et al.*, 2015; Barbut *et al.*, 2024)
  - Species: *Arabidopsis thaliana,* rice (*Oryza sativa)*, multiple species
- **Phosphate**
  - Stress response function: Phosphate status regulates drought, salinity, and temperature stress responses via the phosphate starvation response (PSR) network (SPX–PHR1, InsP8); PHO1 intersects ABA signaling to modulate stomata under salinity/drought; early Pi stress triggers systemic vascular mRNA signals; extensive cross‑talk with other stresses (Satheesh *et al.*, 2022; Khan *et al.*, 2023)
  - Species: *Arabidopsis thaliana*, multiple crops
- **Rhamnose**
  - Stress response function: Component of cell wall polymers; UDP‑L‑rhamnose biosynthesis is crucial for wall integrity; disruptions affect tolerance to acid/oxidative stress and development, underscoring protective roles linked to cell wall function (Jiang *et al.*, 2021).
  - Species: Multiple plant species (dicots and grasses)
- **Betaine (Glycine betaine)**
  - Stress response function: Compatible osmolyte accumulating under abiotic stress; stabilizes membranes and proteins; contributes to ROS management; not all plants synthesize GB at meaningful levels; engineering GB biosynthesis enhances stress resistance (Mäkelä *et al.*, 2019; Hernandez-Leon & Valenzuela-Soto, 2023)
  - Species: Halophytes, *Poaceae* members, engineered crops
- **Fructose**
  - Stress response function: Soluble sugar involved in osmotic adjustment and stress signaling; contributes to “sweet immunity”; accumulates under chilling stress in several species (Wang *et al.*, 2018) (Jeandet *et al.*, 2022)
  - Species: Multiple plant species
- **Ethanolamine**
  - Stress response function: Headgroup of membrane phosphatidylethanolamine; direct, specific stress‑response roles in plants are limited/indirect (Zhang *et al.*, 2023).
  - Species: *Arabidopsis thaliana*
- **Myristic acid**
  - Stress response function: High myristic acid at the sn‑2 position enhances repair of photodamaged PSII under strong light in cyanobacteria; lipid composition/signaling changes are associated with drought responses in plants (Ullah *et al.*, 2022; Kurima *et al.*, 2024)
  - Species: *Synechocystis* (cyanobacteria - photodamage), multiple plant species (lipid signaling context)
- **Ribonic acid**
  - Stress response function: Levels are altered by phosphate stress in tea; linked with GABA and abiotic stress responses in root metabolome studies; unspecific responses (Ding *et al.*, 2017; Schillaci *et al.*, 2021)
  - Species: Tea (*Camellia sinensis*), *Brachypodium*
- **Glycerol‑glycoside**
  - Stress response function: Glycerol can activate defense‑related mechanisms; glycosylation state may affect oxidative stress responses; (Zhang *et al.*, 2015; Lavell & Benning, 2019)
  - Species: Diverse plant species
- **Epigallocatechin (EGCG)**
  - Stress response function: involved in activating the jasmonate pathway (Hong *et al.*, 2015; Li *et al.*, 2019)
  - Species: various species
- **Galactinol**
  - Stress response function: Precursor for raffinose family oligosaccharides (RFOs); acts as compatible solute/antioxidant; overexpression of galactinol synthase is linked to improved abiotic stress tolerance in several species (Jeandet *et al.*, 2022)
  - Species: A*rabidopsis thaliana, Populus trichocarpa, Medicago falcata, citrus*
- **Gluconic acid**
  - Stress response function: can increase as exudate phosphate availability under nutrient limitation (Panchal *et al.*, 2021). No clear metabolic function known in stress response.
  - Species: various (for exudate function)
- **Kaempferol**
  - Stress response function: Flavonol antioxidant contributing to oxidative stress tolerance (Periferakis *et al.*, 2022; Ramaroson *et al.*, 2022)
  - Species: Multiple plant species
- **Meso‑erythritol**
  - Stress response function: Limited evidence for a direct stress‑response role;
  - Species: not applicable
- **Ononitol (D‑ononitol)**
  - Stress response function: Osmoprotectant reported to reduce water loss and confer drought/salt tolerance in soybean and tobacco (Sheveleva *et al.*, 1997; Bartels & Sunkar, 2005).
  - Species: Soybean (*Glycine max*), tobacco (*Nicotiana tabacum*)
- **3-Hydroxypyridine**
  - Stress response function: No direct relationship to plant stress is reported; most literature concerns microbial degradation or human photobiology. (Wondrak *et al.*, 2004; Wang *et al.*, 2020)
  - Species: Not applicable
- **Glycolic acid**
  - Stress response function: redox balance (Messant *et al.*, 2018; Yao *et al.*, 2022)
  - Species: *Arabidopsis thaliana, Solanum tuberosum.*
- **Melezitose**
  - Stress response function: Trisaccharide found in honeydew; no clear direct plant stress‑response role documented.
  - Species: not applicable
- **Palmitic acid**
  - Stress response function: Saturated fatty acid often elevated under drought; involved in membrane adaptation and lipid‑mediated stress signaling (Ullah *et al.*, 2022).
  - Species: multiple species
- **Tetradecane**
  - Stress response function: Herbivore‑induced volatile reported to enhance resistance via jasmonic acid–mediated defenses and accumulation of defensive metabolites (Wang *et al.*, 2021)
  - Species: multiple species
- **Threonic acid**
  - Stress response function: Ascorbate catabolite associated with drought‑adaptive responses in chickpea metabolome studies; linked to ascorbate metabolism in stress contexts(Zhang *et al.*, 2023; Chaturvedi *et al.*, 2024)
  - Species: multiple species

**Table S3.** Transcriptomic response in the offspring of water treated mother trees on different conditions within the experiments with analysis of differentially expressed genes and their functions

| Treatments | Number of genes count | Number of DEGs with p < 0.05 | Number of DEGs with p < 0.01 | GO term: biological process (BP) | | GO term: molecular functions (MF) | |
| --- | --- | --- | --- | --- | --- | --- | --- |
|  |  |  |  | Upregulation | Downregulation | Upregulation | Downregulation |
| Irrigation *vs* control | 15528 | 1370 | 464 | - | Cell killing | - | Antioxidant activity |
| Irrigation *vs* control under heat and drought | 15975 | 690 | 253 | - | Cell killing | - |  |
| Irrigation *vs* control under ambient temperature and water | 15948 | 666 | 218 | - | Immune system process | Electron transfer activity and nutrient reservoir activity | Antioxidant activity and nutrient reservoir activity |
| Irrigation-stop *vs* control | 15570 | 990 | 293 | Copper ion export | - | - | - |
| Irrigation-stop *vs* control under heat and drought | 15756 | 727 | 276 | Immune system process | - | Antioxidant activity an electron transfer activity | - |
| Irrigation-stop *vs* control under ambient temperature and water | 16023 | 426 | 166 | - | - | Antioxidant activity | Electron transfer activity |

**Table S4. Extended Results of Gene expression**

**Genes and transcription factors involved in drought stress responses.**

We analyzed genes essential for drought stress pathways, focusing on water deficit stress (WDS) induced and abscisic acid (ABA)- dependent pathways. ABA is crucial for hormone production and osmotic signaling during drought. We also examined late embryogenesis (LEA) genes, dehydration-induced genes, and those coding for proteins involved in osmolyte synthesis, detoxification, and chaperones, all contributing to stress tolerance. Additionally, we assessed Ca^2+^-related genes, recognizing Ca^2+^ as key for signaling stress onset and activating tolerance mechanisms. Lastly, we included protein factors that regulate gene expression, such as various transcription factors, including DRE-binding protein (DREB), ethylene-responsive element binding factor (ERF), zinc-finger, WRKY, MYB, bHLH, bZIP, NAC, and homeodomain families.

From 15 gene models of ABA/WDS proteins influenced by WDS, ABA, and ripening, we identified 11 genes whose expression changed in progenies of mother trees under different irrigation regimes (Supplementary Excel E1-P1). Expression changes were noted in seedlings under ambient conditions with varying temperature and water. PtASR1 (PITA_23545, Abscisic acid, stress, ripening-induced-like) uniquely showed opposite regulation patterns between treatments; it was upregulated in progenies from irrigated trees and downregulated in those from irrigation-stop trees across all seedling treatments (Supplementary Excel E1-P1).

Of the 67 expressed LEA (late embryogenesis abundant) proteins, 26 were DEGs (differentially expressed genes) across all seedling treatments. Among these genes, 8 genes were up- and 2 downregulated in irrigation (IR) progenies across all treatments. Three genes were upregulated under drought conditions in IR progenies and downregulated in IS progenies (PITA_34172, PITA_31073, and PITA_16807), and two upregulated both in IR and IS (PITA_34470 and PITA_43866) (Supplementary Excel E1-P2). Dehydration-related genes were expressed at relatively low levels in all treatments, and most did not change expression in response to the seedling treatment.

Of the 195 annotated Ca^2+^-related proteins, 40 were differentially expressed in any of the treatments (Supplementary Excel E1-P3). From these, we focus on 10 stable expression patterns between IR and IS progenies under heat and drought (Supplementary Excel E1-P3). Four transcripts, including 3 CBPs (calcium-binding proteins) and 1 CPK (calcium-dependent protein kinases) were upregulated in IR and downregulated in IS. Three transcripts, 2 PMCA (Ca^2+^ ATPases) and one CPK (PITA_31312, PITA_06846, PITA_29659) were upregulated only in IS and two transcripts (PITA_40819, PITA_38357) were downregulated in both IR and IS (Supplementary Excel E1-P3).

From more than 1130 transcription factors (TF) expressed in the analyzed samples, we selected 238 differentially expressed in at least one treatment. 10 TFs were upregulated both in IR and IS progenies under heat and drought conditions, including ethylene-responsive transcription factors, ERF-like, MYB-like, bHLH-like, TCP-like and TRY-like factors. There were 64 TFs upregulated in IR and not changed or downregulated in IS. In contrary, there were only 19 TFs upregulated in IS and not altered or downregulated in IR (Supplementary Excel E1-P4)

**Epigenetic mechanisms related genes.**

Despite a clear lack of epigenetic memory shown on the phenotypic and metabolic level in the seedlings of mother trees originating from different irrigation treatments, we analysed the expression of specific genes involved in epigenetic mechanisms in pine seedlings.

*Methylation and acetylation*

One of the most well-studied epigenetic mechanisms is DNA methylation. In total, we found 17 gene models containing the C5 cytosine-specific DNA methylase domain (Table 4). Six genes were similar to MET2 and DNMT2 (DNA methylases), seven showed similarities to CMT (Chromomethylase) genes, and three to DRMs (Domains Rearranged Methyltransferases). From them, just seven genes changed their expression in response to any treatments. Only two genes were upregulated in heat conditions in IR and IS progenies. All others were downregulated at different treatments (Supplementary Excel E2-P1).

Methylation of histones at lysine and arginine residues is another central epigenetic mechanism and is catalyzed by histone methyltransferases (HMT), lysine methyltransferases (KMT) and arginine methyltransferases (PRMTs) (Supplementary Excel E2-P2). Most KMTs possess a SET domain, Pre-SET motif or SAD(YDG)/SRA domain. Among 113 histone methyltransferase gene models, 11 were DEGs and two of them were stably upregulated for IR and one was downregulated for IS. 45 genes showed similarity to the lysine-specific demethylase JMJ25-like and could be involved in histone demethylation. Six of them were DEGs in any of treatments and one of them – jmjC domain-containing protein 4 (PITA_12617) was DEG in the progenies of IS mother trees and IR mothers in strong drought conditions (Supplementary Excel E2-P3).

Histones can be reversibly acetylated on several lysine residues. Histone acetylation involves activity of histone acetyl- transferases (HATs) and deacetylases (HDACs). There are three groups of HATs: GNAT family acetyltransferase), MYST-like histone acetyltransferases and histone acetyl- transferases subunit 3. Among the 49 HAT genes, only 9 were DEGs. Four HAT protein encoding genes showed upregulation in IR and only one in IS. Among the 31 HDAC-complex genes, only four DEGs were found with inconsistent differences for some treatments (Supplementary Excel E2-P4).

Chromatin remodelers alter the nucleosome composition and positions in the chromatin structure, which is crucial for controlling DNA accessibility and hence regulation of gene expression. From 125 putative chromatin remodelers, we found 28 DEGs changing their expression under different treatments (Table 4). From them, 8 genes were inconsistently upregulated in IR, and 5 genes were inconsistently upregulated in IS (Supplementary Excel E2-P5).

*Modified DNA and chromatin binding protein genes (epigenetic readers)*

MBD (Methyl-CpG-binding domain) proteins are key readers of DNA methylation. They function as interpreters of DNA methylation by recognising and binding to methylated CpG sites in DNA and recruiting chromatin remodelers and other modifying enzymes. Of 37 genes with an MBD domain (Table 4), 10 were DEGs at least in one treatment. From the DEGs, two genes were upregulated in IS and one in IR progenies (Supplementary Excel E2-P6).

PHD (plant homeodomain) finger proteins contain zinc finger motifs that often recognize specific methylated lysine residues, typically associated with active transcription. PHD fingers are encoded by 102 genes, of which 12 were differentially expressed in any of the treatments. One of them was upregulated in IR under heat stress. Another gene (PITA_17418), which is similar to JADE1 protein, that plays a key role in HBO1 complex by directing KAT7/HBO1 specificity towards histone H4 acetylation regulating DNA replication initiation was downregulated in IR (Supplementary Excel E2-P7).

Several PWWP (Pro-Trp-Trp-Pro) domain-containing proteins have been identified. These proteins can bind to methylated histones and are involved in regulating gene expression, DNA repair, and other chromatin-related processes. Here, one DEG gene was found to be upregulated in IR under heat stress (Supplementary Excel E2-P7).

There are 59 genes of proteins containing Chromo (CHRromatin Organisation MOdifier) domain, from which 7 were DEGs in any of the treatments, one of them was upregulated in IS under heat, and one was upregulated in IR under heat treatment. Amongst 48 genes coding for bromodomain-containing proteins, which bind to acetylated lysine residues on histone tails and regulate gene expression, six DEGs were found, of which four had the highest transcript levels in IR (Supplementary Excel E2-P7).

From 16 BRCT domain proteins which are invovlved in post-translational modification of proteins, Poly(ADP-ribosyl)ation of histones and the regulation of chromatin structure, 5 genes were differentially expressed. Two of them, representing Poly (ADP-ribose) polymerase and DNA repair protein REV1-like, were upregulated in IR under heat. We found more than 450 WD40-containing proteins, which are essential for transcriptional regulation and involved in chromatin modification and histone methylation. 29 of them were upregulated in IR and 12 in IS, mostly under heat stress (Supplementary Excel E2-P7).

*miRNAs and other sRNAs biogenesis pathway genes*

sRNAs and miRNAs, in particular, are among the main epigenetic players. We did not analyse sRNAs in our study but examined the transcript profiles for homologs involved in processing, maturation and targeting of miRNAs in plants. From 42 DICER-like genes, only one was upregulated both in IR and IS and another one was downregulated in IR (AGO in Supplementary Excel E2-P8). We found 41 ARGONAUTE-like genes (AGO in Supplementary Excel E2-P9), six of which were differentially expressed in any treatment. Three genes were upregulated; two of them were upregulated in IS and one in IR under heat stress. In addition to PAZ genes, we found 43 genes, considered to be involved in miRNA biogenesis. Only three of them were DEGs, showing differential expression in any of treatments (Supplementary Excel E2-P8 and E2-P9).

The RNA-directed DNA methylation (RdDM) pathway is a plant-specific epigenetic mechanism that establishes de novo DNA methylation using small interfering RNAs (siRNAs). It plays crucial roles in silencing transposable elements (TEs), regulation of gene expression and genomic imprinting. There are several groups of genes involved in the pathway (Supplementary Excel E2-P10).

From three differentially regulated RDRs (RNA dependent RNA polymerase) transcripts, one was upregulated in IR and another was upregulated in IS, both under heat stress. From 25 SHH (SAWADEE HOMEODOMAIN HOMOLOGs)-like transcripts only one was upregulated in IR under heat stress. From 70 POL-like transcripts, we found two genes were consistently upregulated in IR and two upregulated in IS. From 33 transcripts with XH/XS domains, seven were upregulated in IR and two were upregulated both in IR and IS (Supplementary Excel E2-P10).

**Figure S1.** **Temporal variation in soil volumetric water content under the watering and temperature treatments.** Points and lines represent mean soil volumetric water content (VWC) for seedlings grown under ambient or elevated temperature (ambient + 4°C) in the well-watered and drought treatments. Shaded bands indicate ± SE. The horizontal dotted line indicates the approximate 10% VWC target of the drought treatment. Soil VWC was measured two to three times per week from 4 July to 7 October 2022. The temporal variation reflects repeated soil-drying and partial-rewetting cycles under the intermittent drought treatment.

**Figure S2.** **Daily mean air temperature across the six climate chambers during the experiment.** Each point represents the daily mean calculated from air-temperature measurements recorded at 10-min intervals within an individual climate chamber. Blue solid lines represent the three ambient chambers (C5.1–C5.3), and warm-colored dashed lines represent the three heated chambers (C5.4–C5.6). Temperature records from 1 July to 7 October 2022 are shown.

**Figure S3.** NSC concentrations across different plant organs and mother tree groups experienced drought and temperature treatments. Error bars represent Mean ± SE.

**Figure S4.** Correlation clusters for similarities within the three groups of mother trees: Memory ‘C’, ‘IR’, and ‘IS’ indicate offspring from control, irrigation and irrigation-stop mother trees. Environment ‘Con’ and ‘Dro’ indicate the control (well-watered) and drought treatments applied to offspring from three different mother trees in the climate chamber.

**References**

Barbut FR, Cavel E, Donev EN, Gaboreanu I, Urbancsok J, Pandey G, Demailly H, Jiao D, Yassin Z, Derba-Maceluch M, Master ER, Scheepers G, Gutierrez L, Mellerowicz EJ. 2024. Integrity of xylan backbone affects plant responses to drought. *Front Plant Sci* 15: 1422701.

Bartels D, Sunkar R. 2005. Drought and Salt Tolerance in Plants. *Critical Reviews in Plant Sciences* 24: 23-58.

Chaturvedi P, Pierides I, López-Hidalgo C, Garg V, Zhang S, Barmukh R, Bellaire A, Li J, Bachmann G, Valledor L, Varshney RK, Ghatak A, Weckwerth W. 2024. Natural variation in the chickpea metabolome under drought stress. *Plant Biotechnol J* 22: 3278-3294.

Ding Z, Jia S, Wang Y, Xiao J, Zhang Y. 2017. Phosphate stresses affect ionome and metabolome in tea plants. *Plant Physiol Biochem* 120: 30-39.

Hernandez-Leon SG, Valenzuela-Soto EM. 2023. Glycine Betaine is a Phytohormone-Like Plant Growth and Development Regulator Under Stress Conditions. *Journal of Plant Growth Regulation* 42: 5029-5040.

Hong G, Wang J, Hochstetter D, Gao Y, Xu P, Wang Y. 2015. Epigallocatechin-3-gallate functions as a physiological regulator by modulating the jasmonic acid pathway. *Physiologia Plantarum* 153: 432-439.

Ibrahim EA. 2025. Review: Trehalose and its role in plant adaptation to salinity stress. *Plant Science* 357: 112533.

Jeandet P, Formela-Luboińska M, Labudda M, Morkunas I. 2022. The Role of Sugars in Plant Responses to Stress and Their Regulatory Function during Development. *Int J Mol Sci* 23.

Jiang N, Dillon FM, Silva A, Gomez-Cano L, Grotewold E. 2021. Rhamnose in plants - from biosynthesis to diverse functions. *Plant Science* 302: 110687.

Khan F, Siddique AB, Shabala S, Zhou M, Zhao C. 2023. Phosphorus Plays Key Roles in Regulating Plants' Physiological Responses to Abiotic Stresses. *Plants (Basel)* 12.

Kurima K, Jimbo H, Fujihara T, Saito M, Ishikawa T, Wada H. 2024. High Myristic Acid in Glycerolipids Enhances the Repair of Photodamaged Photosystem II under Strong Light. *Plant and Cell Physiology* 65: 790-797.

Lavell AA, Benning C. 2019. Cellular Organization and Regulation of Plant Glycerolipid Metabolism. *Plant and Cell Physiology* 60: 1176-1183.

Li X, Li Y, Ahammed GJ, Zhang X-N, Ying L, Zhang L, Yan P, Zhang L-P, Li Q-Y, Han W-Y. 2019. RBOH1-dependent apoplastic H2O2 mediates epigallocatechin-3-gallate-induced abiotic stress tolerance in Solanum lycopersicum L. *Environmental and Experimental Botany* 161: 357-366.

Mäkelä PSA, Jokinen K, Himanen K 2019. Roles of Endogenous Glycinebetaine in Plant Abiotic Stress Responses. In: M. A. Hossain, V. Kumar, D. J. Burritt, M. FujitaP. S. A. Mäkelä eds. *Osmoprotectant-Mediated Abiotic Stress Tolerance in Plants: Recent Advances and Future Perspectives*. Cham: Springer International Publishing, 153-173.

Messant M, Timm S, Fantuzzi A, Weckwerth W, Bauwe H, Rutherford AW, Krieger-Liszkay A. 2018. Glycolate Induces Redox Tuning Of Photosystem II in Vivo: Study of a Photorespiration Mutant. *Plant Physiol* 177: 1277-1285.

Panchal P, Miller AJ, Giri J. 2021. Organic acids: versatile stress-response roles in plants. *Journal of Experimental Botany* 72: 4038-4052.

Periferakis A, Periferakis K, Badarau IA, Petran EM, Popa DC, Caruntu A, Costache RS, Scheau C, Caruntu C, Costache DO. 2022. Kaempferol: Antimicrobial Properties, Sources, Clinical, and Traditional Applications. *Int J Mol Sci* 23.

Ramaroson M-L, Koutouan C, Helesbeux J-J, Le Clerc V, Hamama L, Geoffriau E, Briard M. 2022. Role of Phenylpropanoids and Flavonoids in Plant Resistance to Pests and Diseases. *Molecules* 27: 8371.

Raza A, Bhardwaj S, Atikur Rahman M, García-Caparrós P, Habib M, Saeed F, Charagh S, Foyer CH, Siddique KHM, Varshney RK. 2024. Trehalose: A sugar molecule involved in temperature stress management in plants. *The Crop Journal* 12: 1-16.

Satheesh V, Tahir A, Li J, Lei M. 2022. Plant phosphate nutrition: sensing the stress. *Stress Biol* 2: 16.

Schillaci M, Kehelpannala C, Martinez-Seidel F, Smith PMC, Arsova B, Watt M, Roessner U. 2021. The Metabolic Response of Brachypodium Roots to the Interaction with Beneficial Bacteria Is Affected by the Plant Nutritional Status. *Metabolites* 11.

Sheveleva E, Chmara W, Bohnert HJ, Jensen RG. 1997. Increased Salt and Drought Tolerance by D-Ononitol Production in Transgenic Nicotiana tabacum L. *Plant Physiol* 115: 1211-1219.

Shi XB, Yue H, Wei Y, Preisser EL, Wang P, Du J, Xia JX, Li KL, Yang X, Chen JB, Zhang SB, Zhang ZH, Zhou XG, Zhang DY, Liu Y. 2025. Neophytadiene, a Plant Specialized Metabolite, Mediates the Virus-Vector-Plant Tripartite Interactions. *Adv Sci (Weinh)* 12: e2416891.

Takano S, Matsuda S, Funabiki A, Furukawa J, Yamauchi T, Tokuji Y, Nakazono M, Shinohara Y, Takamure I, Kato K. 2015. The rice RCN11 gene encodes β1,2-xylosyltransferase and is required for plant responses to abiotic stresses and phytohormones. *Plant Sci* 236: 75-88.

Ullah S, Khan MN, Lodhi SS, Ahmed I, Tayyab M, Mehmood T, Din IU, Khan M, Sohail Q, Akram M. 2022. Targeted metabolomics reveals fatty acid abundance adjustments as playing a crucial role in drought-stress response and post-drought recovery in wheat. *Front Genet* 13: 972696.

Wang H, Gong M, Xin H, Tang L, Dai D, Gao Y, Liu C. 2018. Effects of chilling stress on the accumulation of soluble sugars and their key enzymes in Jatropha curcas seedlings. *Physiology and Molecular Biology of Plants* 24: 857-865.

Wang H, Wang X, Ren H, Wang X, Lu Z. 2020. 3-Hydroxypyridine Dehydrogenase HpdA Is Encoded by a Novel Four-Component Gene Cluster and Catalyzes the First Step of 3-Hydroxypyridine Catabolism in Ensifer adhaerens HP1. *Applied and Environmental Microbiology* 86.

Wang Y, Mostafa S, Zeng W, Jin B. 2021. Function and Mechanism of Jasmonic Acid in Plant Responses to Abiotic and Biotic Stresses. *Int J Mol Sci* 22.

Wondrak GT, Roberts MJ, Jacobson MK, Jacobson EL. 2004. 3-hydroxypyridine chromophores are endogenous sensitizers of photooxidative stress in human skin cells. *Journal of Biological Chemistry* 279: 30009-30020.

Yao Z, Rao Z, Hou S, Tian C, Liu CY, Yang X, Zhu G. 2022. The appropriate expression and coordination of glycolate oxidase and catalase are vital to the successful construction of the photorespiratory metabolic pathway. *Front Plant Sci* 13: 999757.

Zhang Y, Smith P, Maximova SN, Guiltinan MJ. 2015. Application of glycerol as a foliar spray activates the defence response and enhances disease resistance of Theobroma cacao. *Molecular Plant Pathology* 16: 27-37.

Zhang Y, Xu J, Li R, Ge Y, Li Y, Li R. 2023. Plants' Response to Abiotic Stress: Mechanisms and Strategies. *Int J Mol Sci* 24.
